# Microtubins: a novel class of small synthetic microtubule targeting drugs that inhibit cancer cell proliferation

**DOI:** 10.18632/oncotarget.21945

**Published:** 2017-10-19

**Authors:** Silvia Senese, Yu-Chen Lo, Ankur A. Gholkar, Chien-Ming Li, Yong Huang, Jack Mottahedeh, Harley I. Kornblum, Robert Damoiseaux, Jorge Z. Torres

**Affiliations:** ^1^ Department of Chemistry and Biochemistry, University of California, Los Angeles, CA 90095, USA; ^2^ Department of Bioengineering, University of California, Los Angeles, CA 90095, USA; ^3^ Drug Studies Unit, Department of Bioengineering & Therapeutic Sciences, University of California, San Francisco, CA 94143, USA; ^4^ Department of Molecular and Medical Pharmacology, Los Angeles, CA 90095, USA; ^5^ Department of Psychiatry, University of California, Los Angeles, CA 90095, USA; ^6^ The Semel Institute for Neuroscience and Human Behavior, University of California, Los Angeles, CA 90095, USA; ^7^ Jonsson Comprehensive Cancer Center, University of California, Los Angeles, CA 90095, USA; ^8^ California NanoSystems Institute, University of California, Los Angeles, CA 90095, USA; ^9^ Molecular Biology Institute, University of California, Los Angeles, CA 90095, USA

**Keywords:** cell division, microtubules, cell cycle, tubulin-targeting agents, cancer cell proliferation

## Abstract

Microtubule targeting drugs like taxanes, vinca alkaloids, and epothilones are widely-used and effective chemotherapeutic agents that target the dynamic instability of microtubules and inhibit spindle functioning. However, these drugs have limitations associated with their production, solubility, efficacy and unwanted toxicities, thus driving the need to identify novel antimitotic drugs that can be used as anticancer agents. We have discovered and characterized the Microtubins (Microtubule inhibitors), a novel class of small synthetic compounds, which target tubulin to inhibit microtubule polymerization, arrest cancer cells predominantly in mitosis, activate the spindle assembly checkpoint and trigger an apoptotic cell death. Importantly, the Microtubins do not compete for the known vinca or colchicine binding sites. Additionally, through chemical synthesis and structure-activity relationship studies, we have determined that specific modifications to the Microtubin phenyl ring can activate or inhibit its bioactivity. Combined, these data define the Microtubins as a novel class of compounds that inhibit cancer cell proliferation by perturbing microtubule polymerization and they could be used to develop novel cancer therapeutics.

## INTRODUCTION

Most anticancer drugs perturb the proliferation cycle of tumor cells and two major classes are those that inhibit the DNA replication cycle (such as DNA damaging agents) and those that inhibit cell division (antimitotics like microtubule poisons). Antimitotics are a group of natural and synthetic small molecules that perturb the functioning of the mitotic microtubule spindle during cell division. Current antimitotics work through binding and inhibition of three major classes of molecules; microtubules, kinases, and kinesins [1]. For example, GSK-461363, a polo like kinase 1 (Plk1) ATP-competitive inhibitor, blocks Plk1-dependent centrosome maturation, which arrests cells in prophase with a monopolar spindle [2]. Similarly, Ispinesib, an allosteric inhibitor of Kinesin-5 arrests cells with a monopolar spindle, due to the inability of Kinesin-5 to separate centrosomes to opposite ends of the cell [3]. Microtubule targeting agents including stabilizers (taxanes like paclitaxel (taxol) and epothilones) and destabilizers (vinca alkaloids and colchicine) bind to tubulin and perturb microtubule dynamics by stabilizing or destabilizing microtubules and thereby their ability to align and segregate chromosomes [4]. Antimitotics activate the spindle assembly checkpoint (SAC), which arrests cells in mitosis until proper microtubule-kinetochore attachment occurs [5, 6]. A prolonged mitotic arrest then activates an apoptotic response leading to cell death [7]. This process occurs through p38, JNK, and CKII kinase mediated phosphorylation of Mcl1, which targets Mcl1 for ubiquitination by the SCF-Fbw7 ubiquitin ligase and proteosome-dependent degradation [1, 7, 8]. Mcl1 destruction relieves its inhibition of Bax and Bak (pro-apoptotic factors), allowing them to bind the mitochondrial outer membrane to induce an apoptotic cell death [1, 7, 8]. Additionally, a prolonged mitotic arrest can lead to DNA damage, which activates a DNA damage response that is p53-dependent leading to subsequent apoptosis [9, 10]. However, cancer cells often misregulate their cell cycle checkpoints and can have varied responses to antimitotics; not only between different types of cancers but also within the same type of cancer cells [5]. Therefore, cancer cells treated with antimitotics may bypass the SAC and undergo an aberrant division, which can later lead to apoptosis from any cell cycle phase [11]. Additionally, it is now well established that the cytotoxic effect of antimitotics is in part due to the disruption of interphase cytoskeletal microtubules [12]. Thus, the effect of antimitotics on cancer cells goes beyond their ability to inhibit cell division.

Although microtubule targeting agents are some of the most common chemotherapeutic agents used to treat a wide variety of cancers, they show important dose-limiting toxicities, including neutropenia and neurotoxicity, largely a consequence of disturbing microtubule dynamics in neurons [13, 14]. Most of the microtubule targeting agents used clinically are large, natural (difficult to synthesize), hydrophobic compounds with limited solubility. In addition, some cancers acquire resistance to these agents by overexpressing drug efflux pumps like MDR1 and MRP1 that lower the intracellular drug concentration, by mutating key amino acids in βI-tubulin that inhibit microtubule targeting drug binding, or by overexpressing βIII-tubulin that is not a target of most microtubule targeting drugs [15]. Thus, there is a critical need to identify novel tubulin-targeting drugs with improved properties that can be used as anticancer agents.

Here, we have discovered and characterized (E)-2-styryl-5,6,7,8-tetrahydrobenzo [4, 5] thieno[2,3-d]pyrimidin-4(3H)-one (Microtubin-1) and its analogues (Microtubins); a novel class of drug-like microtubule targeting agents that inhibit cancer cell proliferation. The Microtubins disrupt microtubule polymerization, arrest cells in mitosis, activate the spindle assembly checkpoint and trigger an apoptotic cell death. Importantly, the Microtubins do not compete with vinblastine for the known vinca binding site or colchicine for the colchicine binding site and inhibit tubulin polymerization through a different mechanism. Microtubin structure-activity relationship (SAR) studies indicated that modification of the Microtubin phenyl ring was critical to modulating its ability to inhibit microtubule polymerization, formation of the mitotic spindle, and cell division. Additionally, the Microtubins were not only active against a cervical adenocarcinoma cell line, but also patient derived glioblastoma cells and multi-drug resistant small cell lung carcinoma cells. Thus, the Microtubins represent a novel class of compounds that could be developed for therapeutic use in the treatment of cancer.

## RESULTS

### Identification of microtubin-1 from a cell-based high-throughput small molecule screen

**Figure 1:**
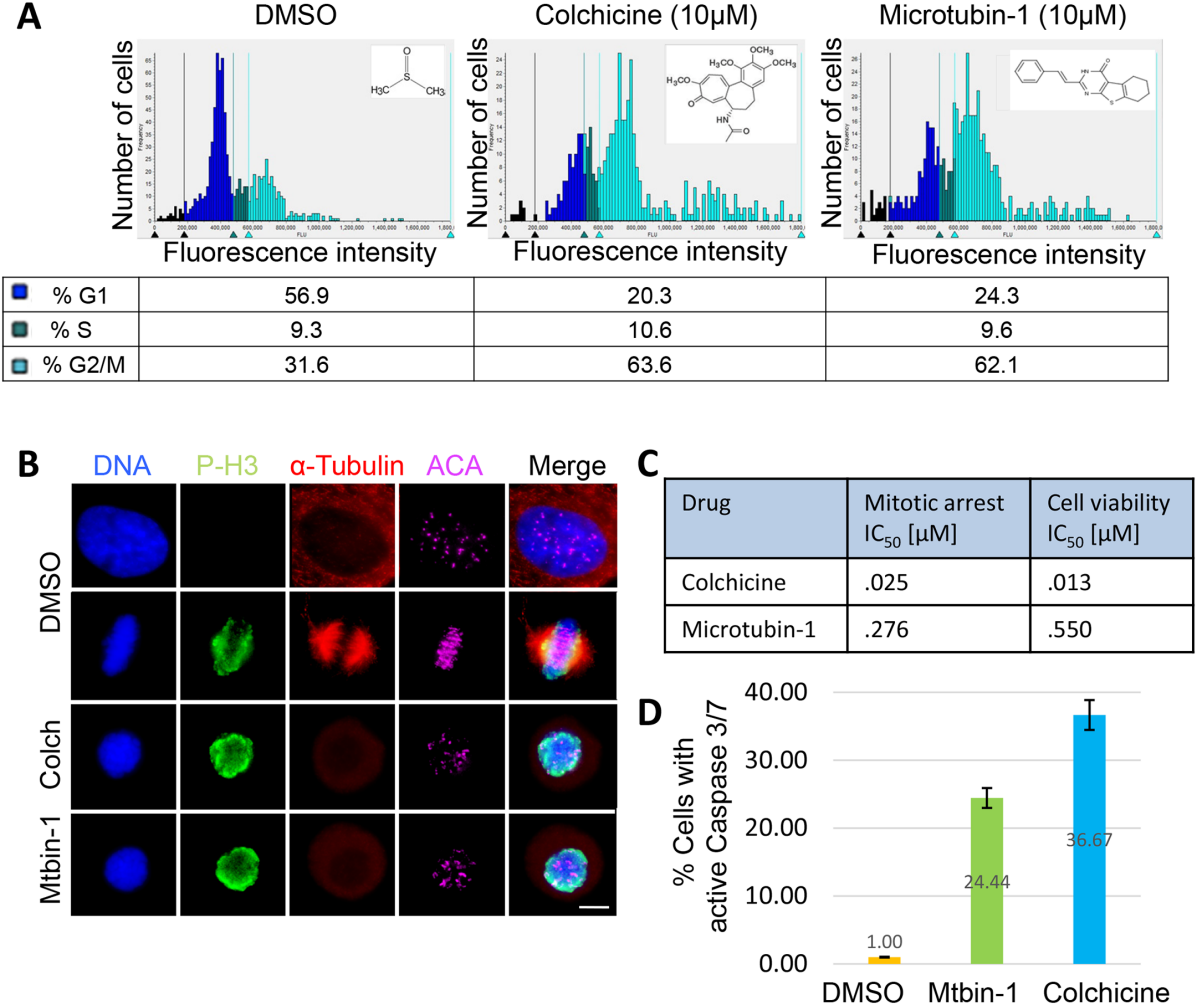
Identification of Microtubin-1, a novel cell division inhibitor **(A)**, cell cycle histogram of HeLa cells treated with DMSO or 10 μM of either colchicine or Microtubin-1 for 20 hours. The chemical structures of DMSO, colchicine and Microtubin-1 are indicated next to their histograms. The percentage of cells in G1 phase, S phase and G2/M phase are indicated below the histogram for each treatment. **(B)**, immunofluorescence microscopy of cells treated with DMSO or 10 μM of either colchicine or Microtubin-1 for 20 hours. Cells were fixed with 4% paraformaldehyde stained with Hoechst 33342 and anti-ɑ-tubulin and anti-Ser10-phospho-histone H3 antibodies to visualize the DNA, microtubule structures and mitotic cells, respectively. Bar indicates 5 μm. **(C)**, HeLa cells were treated with increasing concentrations of colchicine and Microtubin-1 and the drug response dose curves were used to measure the mitotic arrest IC_50s_ (Vybrant DyeCycle Green assay) and the cell viability IC_50s_ (CellTiter-Glo assay) for each treatment. **(D)**, HeLa cells were treated with DMSO, colchicine and Microtubin-1 for 24 hours and caspase 3/7 activity was measured using the Caspase-Glo 3/7 assay. Graph displays % cells undergoing apoptosis (with active caspase 3/7) on the y-axis and the indicated drug treatments on the x-axis. Error bars indicate standard deviations from 3 independent triplicate experiments.

To discover novel compounds that inhibit cancer cell proliferation, we recently performed a cell-based high-throughput small molecule screen with a diverse library of 79,827 compounds covering broad chemical space containing drug-like molecules (at a final [10 μM]) for their ability to arrest cancer cell division [16]. Briefly, Human cervical adenocarcinoma (HeLa) cells were treated with DMSO or one of the 79,827 compounds in the library for twenty hours and their cell cycle profile was analyzed using the DNA-selective stain Vybrant DyeCycle Green; a cell membrane permeant dye that binds to DNA and emits a fluorescent signal that is proportional to DNA mass when excited at 488 nm with a cytometer [17]. This approach yielded (E)-2-styryl-5,6,7,8-tetrahydrobenzo [4, 5] thieno[2,3-d]pyrimidin-4(3H)-one (Microtubin-1) (Figure 1A). Microtubin-1 is a small (308.4 Da) synthetic compound with predicted drug-like properties [18–20] (Table 1). The cell cycle histogram profiles showed that similar to colchicine, Microtubin-1 was able to arrest the majority of the cells in G2/M phase (63.6% for colchicine and 62.1% for Microtubin-1, compared to control 31.6%) (Figure 1A).

**Table 1:** Predicted chemical properties of Microtubins and other microtubule targeting agents

In-silico Chemical Properties Predictions	Ideal	Mtbin-1	Mtbin-2	Mtbin-3	Vinblastine	Vincristine	Colchicine
BBB permeability (Clark et al.)^a^	yes	yes	yes	yes	no	no	no
Lipophilicity (logP)	∼4	4.01	4.35	4.35	4.34	3.58	2.2
H-bond donors	<7	1	1	1	4	5	1
Polar surface area (PSA), A^o^	40 – 90	82.7	81.43	81.39	189.9	215.6	83
Molecular weight	∼400	308.4	344.4	344.4	853.9	807.9	507.7
Oral bioavailability (Veber Rules)^b^	yes	yes	yes	yes	no	no	yes
Rotatable bonds	<10	2	2	2	15	14	6
Polar surface area (PSA), A^o^	<140	82.7	81.43	81.39	189.9	215.6	83
Absorption/permeability (Lipinski Rules)^c^	yes	yes	yes	yes	no	no	yes
H-bond donors	<5	1	1	1	4	5	1
Molecular weight, daltons (Da)	<500	308.4	344.4	344.4	853.9	807.9	399.4
logP	<5	4.01	4.35	4.35	4.34	3.58	2.2
H-bond acceptors	<10	3	3	3	15	15	7
Solubility (logS)	> -5.7	−5.519	−6.1	−6.1	−8.43	−7.1	−4.18
Pgp efflux substrate	no	no	no	no	yes	yes	yes
H-bond donors	<8	1	1	1	4	5	1
Molecular weight, daltons (Da)	<400	308.4	344.4	344.4	853.9	807.9	399.4

LogP: lipophilicity, Lipinski rules (HD<5, MW<500, logP<5, HA<10), Veber rules (RotB<=10, PSA<=140A) References: *a* (20), *b*(18), *c* (19).

### Microtubin-1 inhibits cancer cell proliferation by arresting cells in mitosis

To determine whether Microtubin-1-treated cells were arresting in mitosis or G2 phase, we performed immunofluorescence microscopy on cells that had been treated with colchicine or Microtubin-1 for 20 hours. In this assay, cells were fixed, permeabilized and co-stained for DNA (Hoechst 3342 DNA dye), ɑ-tubulin (anti-tubulin antibodies), centromeres (anti-centromere antibodies, ACA), and the mitosis marker p-H3 (anti-phospho-Ser10-histone H3 antibodies). This analysis indicated that colchicine and Microtubin-1-treated cells arrested in mitosis (positive for p-H3) with condensed chromosomes and depolymerized microtubules [21, 22] (Figure 1B). Next, HeLa cells were treated with DMSO or a nineteen point two-fold titration (19 nM to 6.25 μM) of colchicine or Microtubin-1 for 20 hours and the mitotic arrest half maximal inhibitory concentration (IC_50_) was measured using the Vybrant DyeCycle Green assay described above. This analysis revealed that colchicine had a mitotic arrest IC_50_= 25 nM and Microtubin-1 had a mitotic arrest IC_50_= 276 nM (Figure 1C). To determine if Microtubin-1 arrested mitotic cells were dying, we utilized the same drug titration series to treat cells for 72 hours and the cell viability was measured using the CellTiter-Glo luminescent cell viability assay, which measures total ATP levels (indicative of metabolically active cells) using a luminometer at 340 nm wavelength. The cell viability IC_50_ was then quantified. This revealed that colchicine had a cell viability IC_50_= 13 nM and Microtubin-1 had a cell viability IC_50_= 550 nM (Figure 1C). Next, we asked if the Microtubin-1 induced cell death was through caspase dependent apoptosis. To do this, HeLa cells were treated with DMSO, colchicine (100 nM) or Microtubin-1 (550 nM) for 24 hours and caspase 3/7 activity was measured using the Caspase-Glo 3/7 assay. Indeed, similar to the colchicine treatment, Microtubin-1 treatment led to an in increase in the percentage of cells with active caspase 3/7 activity compared to the DMSO control, 24.4% and 36.7% respectively (Figure 1D). Together these results indicated that Microtubin-1 was inhibiting microtubule polymerization, which arrested cells in mitosis and activated an apoptotic cell death to decrease the viability of cervical adenocarcinoma cells.

### Microtubin-1 does not compete for the known vinca or colchicine tubulin sites

**Figure 2:**
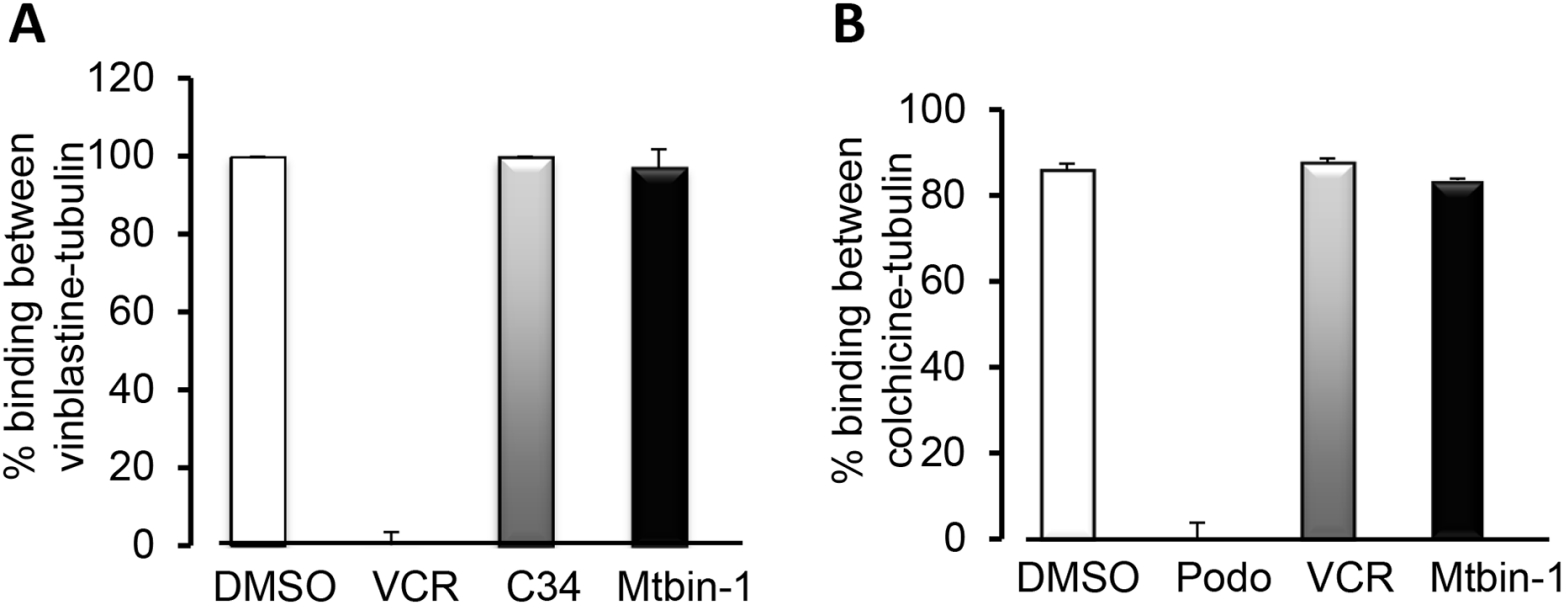
Microtubin-1 does not compete for binding to the vinca-binding site or the colchicine-binding site **(A-B)**, mass spectrometry-based competitive binding assays to test the binding of Microtubin-1 (Mtbin-1) to the vinca (A) and colchicine (B) site. All compounds were tested at 100 μM. Graphs display % binding between vinblastine and tubulin (A) or colchicine and tubulin (B) on the y-axis and the indicated drugs used to compete the binding on the x-axis. Data represent the average ± SD. A, Microtubin-1 does not compete with vinblastine for binding to the vinca site compared to the positive control vincristine (VCR). C34 is the negative control compound 34. B, Microtubin-1 does not compete with colchicine for the colchicine site compared to the positive control podophyllotoxin (Podo).

The mechanism of action for microtubule depolymerizing agents can be classified on the basis of where they bind to within tubulin, which include the vinca site (bound by large natural compounds like the vinca alkaloids vincristine and vinblastine) and the colchicine site (bound by small compounds like colchicine and podophyllotoxin) [23, 24]. Thus, we used a mass spectrometry-based competition assay to determine if Microtubin-1 was binding to either of these two sites or to a novel site [25, 26]. First, we analyzed whether Microtubin-1 was able to compete the vinblastine-tubulin interaction compared to vincristine, which binds to the vinca site. This analysis showed that Microtubin-1 was not able to compete the vinblastine-tubulin interaction similar to a negative control compound 34 (C34), whereas vincristine (VCR) was able to compete this interaction (Figure 2A). Similarly, we analyzed the ability of Microtubin-1 to compete the colchicine-tubulin interaction compared to podophyllotoxin, which binds the colchicine site. Interestingly, Microtubin-1 was also not able to compete this interaction similar to the negative control vincristine (VCR), whereas podophyllotoxin (podo) was able to compete this interaction (Figure 2B). These results indicated that Microtubin-1 was not binding to the vinca or colchicine sites and was likely targeting a novel site.

### Improving microtubin-1 activity

**Figure 3:**
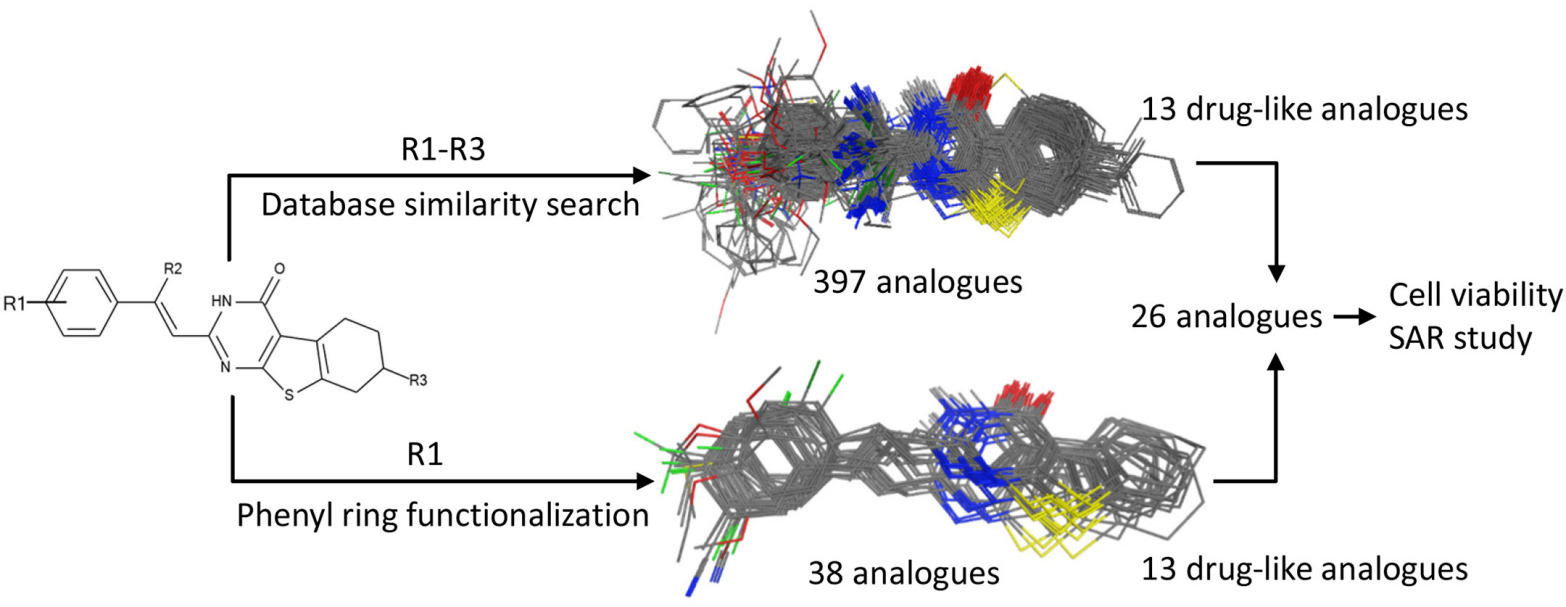
Microtubin-1 optimization A ligand similarity search identified 397 compounds with >80% ligand similarity with Microtubin-1. Additionally, the Topliss scheme for aromatic ring optimization was used to identify 38 Microtubin-1 phenyl ring derivatives. The top 13 drug-like analogues and 13 drug-like phenyl derivatives of Microtubin-1 were selected for testing in HeLa cell culture structure activity relationship studies.

To improve the antiproliferative activity of Microtubin-1 without knowledge of its binding site and to understand the chemical properties that influence Microtubin-1 activity, we took two complementary ligand-based approaches (Figure 3). First, we searched the ZINC compound library to identify Microtubin-1 structural analogues sharing >80% similarity [27]. This approach yielded 397 compounds with varied functional group additions to the core scaffold of Microtubin-1 (Figure 3 and Supplementary Table 1). As an alternative approach, we evaluated the synthetic routes based on the core structure of Microtubin-1 for ease of chemical modification and subsequently designed 38 phenyl ring derivatives based on the Topliss scheme for aromatic ring optimization [28] (Figure 3 and Supplementary Table 2). To further improve the potential of these compounds for cellular activity, we subjected these analogues to a drug-like filter using Lipinski’s rule of five, based on a series of chemical descriptors of known bioactive drugs [29]. We then selected and acquired or synthesized (Supplementary Figure 1) 13 analogues from each group (a total of 26 analogues) with diverse functional modifications for further testing (Figure 3, Table 2 and Supplementary Table 3).

**Table 2:** Cell viability IC_50_ values for Microtubins tested in HeLa cells

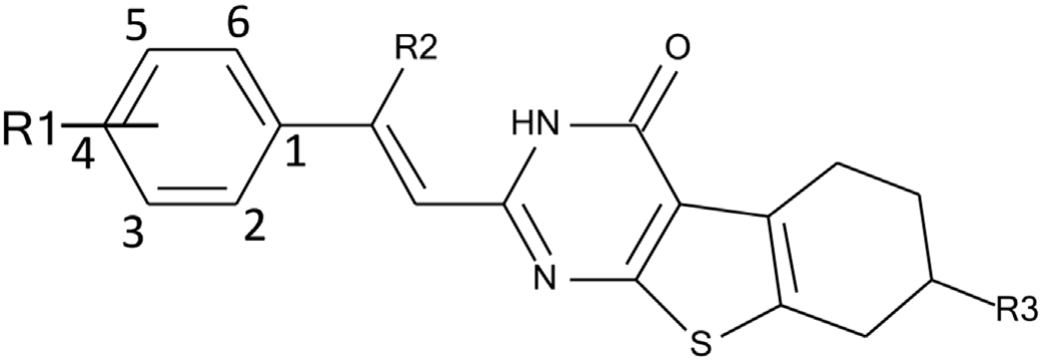
Microtubin	R1	R2	R3	IC_50_[μM]
1	H	H	H	0.552
2	2,4-F	H	H	0.246
3	2,5-F	H	H	0.159
4	4-F	H	H	1.11
5	3-OCH_3_	H	H	4.21
6	4-SCH_3_	H	H	6.31
7	3-OCH_3_,4-OCH_2_CH_3_	H	H	25.9
8	3-CH_3_	H	H	>100
9	4-OCH_3_	H	H	50.9
10	4-Br	H	H	>100
11	3,4-F	H	H	>100
12	3,4-OCH_3_	H	H	>100
13	4-OCH_2_CH_3_	H	H	>100
14	4-CH_3_	H	H	>100
15	4-OCF_3_	CN	3-CH_3_	>100
16	2-OCF_3_	CN	3-CH_3_	>100
17	2,5-CH_3_	CN	3-CH_3_	>100
18	4-CF_3_	CN	H	>100
19	2,6-Cl	CN	3-CH_3_	>100
20	3,4-Cl	H	H, tetra decane ring	>100
21	3-CH_3_	CN	3-CH_3_	>100
22	2-CF_3_	H	H	>100
23	4-CH_2_CH_3_	CN	3-CH_3_	>100
24	2-CH_3_	CN	3-CH_3_	>100
25	2,4,6-CH_3_	CN	H	>100
26	2-CF_3_	CN	H	>100
27	2,4,6-CH_3_	CN	3-CH_3_	>100

### In-cell microtubin structure-activity relationship (SAR) studies

To understand the chemical parameters that were important for the activity of Microtubin-1 and to determine if Microtubin-1 analogues were more potent, we evaluated the 26 Microtubin-1 analogues in cell culture-based structure-activity relationship (SAR) studies. HeLa cells were plated into 384 well plates and treated with a twenty point two-fold titration (24 nM to 100 μM) of each compound, Microtubin-1-27, for 72 hours and cell viability was measured using the CellTiter-Glo luminescent cell viability assay as described previously. The cell viability IC_50_ was then quantified for each compound. From this, we identified three compounds with an IC_50_ in the nanomolar range that included Microtubin-1 (IC_50_ = 552 nM), Microtubin-2 (IC_50_ = 246 nM) and Microtubin-3 (IC_50_ = 159 nM) (Table 2). A summary of the Microtubin SAR is presented in Table 2. The Microtubin SAR indicated that functionalization of the phenyl ring could improve Microtubin activity while substitution at other positions of the core scaffold, including the addition of a nitrile group at the R2 position, the addition of a 3-methyl group at the R3 position or ring expansion of the tricyclic core inactivated the compounds (Table 2). The preferred aromatic ring substitutions included fluorination at the 2, 4, and 5 positions, while methoxy or sulfur methyl substitution at the 3 or 4 positions also led to an observed cytotoxicity (Table 2). The SAR also indicated that the position of the substituents was critical for the activity of these compounds. For example, addition of a 3-methoxyl group to Microtubin-13 activated the compound (Microtubin-7), while further removal of the 4-ethoxy increased potency by 6 fold (Microtubin-5) (Table 2). Among the active compounds (including Microtubin-5, Microtubin-7 and Microtubin-9) the methoxy and its bioisostere sulfur methyl group primarily substituted the 3 and 4 positions of the phenyl ring but not at other positions (Table 2).

### Microtubins inhibit microtubule polymerization

**Figure 4:**
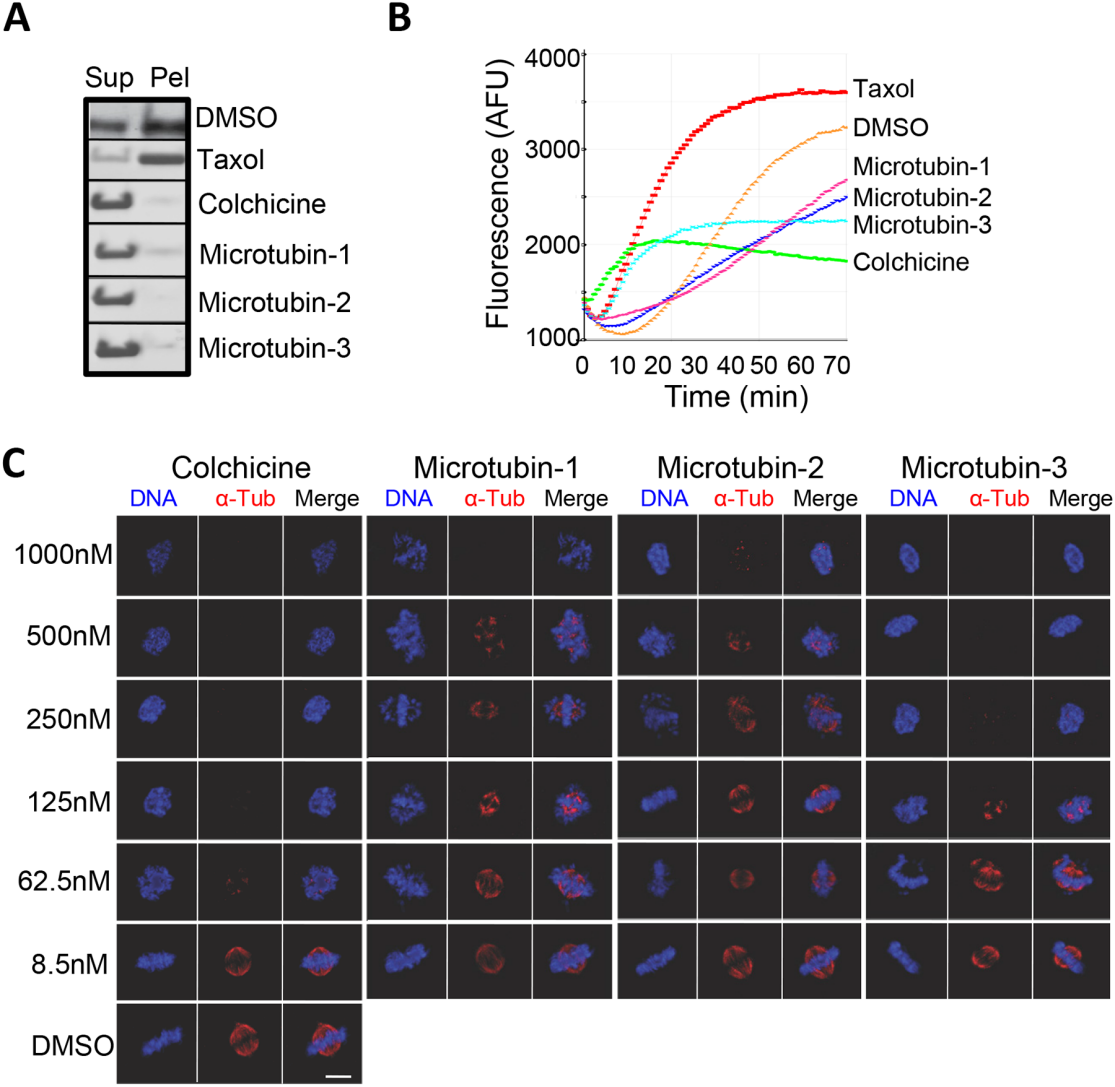
The Microtubins inhibit microtubule polymerization *in vitro* and in cells **(A-B)**, *in vitro* microtubule polymerization reactions were carried out in the presence of DMSO, or 15 μM of taxol, colchicine, Microtubin-1, Microtubin-2, or Microtubin-3 for 70 minutes at 37°C. A, reaction products were subjected to centrifugal sedimentation and the supernatant (Sup) and pellet (Pel) fractions were resolved by SDS-PAGE and tubulin polymerization was visualized with Coomassie blue staining. B, microtubule polymerization was monitored over time every minute by measuring the absorbance at 340 nm. Graph displays fluorescence signal (in arbitrary fluorescence units) on the y-axis over time (in minutes) on the x-axis for the indicated drug treatments. **(C)**, immunofluorescence microscopy of cells treated with DMSO or increasing concentrations of taxol, colchicine, Microtubin-1, Microtubin-2, or Microtubin-3 for 20 hours. Cells were fixed with 4% paraformaldehyde and stained with Hoechst 33342 and anti-ɑ-tubulin antibodies to visualize the DNA and microtubule structures, respectively. Bar indicates 5 μm.

To further validate that Microtubin-1 and its analogues were targeting microtubules, we performed *in vitro* microtubule polymerization reactions with the most potent compounds (Microtubin-1-3) using an *in vitro* microtubule polymerization assay [26]. In this assay, 15 μM Microtubin-1-3, colchicine or taxol were added to the reaction mixture and microtubules were allowed to polymerize at 37°C for 70 minutes. Microtubule polymerization was monitored by endpoint and kinetic measurements. For endpoint measurements, polymerization reactions were subjected to centrifugal sedimentation and the supernatant and pellet fractions were resolved by SDS-PAGE and gels were stained with Coomassie blue to visualize the quantity of polymerized microtubules in the pellet fraction versus non-polymerized tubulin in the supernatant (Figure 4A). For kinetic measurements, microtubule polymerization was monitored by reading the fluorescence at 420 nm (due to the incorporation of a fluorescent reporter into microtubules as polymerization occurred) every minute using a Tecan M1000 microplate reader (Figure 4B). Both endpoint and kinetic measurements indicated that *in vitro* Microtubin-1, Microtubin-2, and Microtubin-3 were inhibitors of microtubule polymerization similar to colchicine (Figure 4A and 4B). Next, we analyzed microtubule stability in HeLa cells treated with increasing concentrations of each of these three compounds. Briefly, HeLa cells were fixed with 4% paraformaldehyde, costained with Hoechst 33342 (DNA dye to visualize their DNA) and anti-ɑ-tubulin antibodies (to visualize microtubule structures) and imaged by immunofluorescence microscopy (Figure 4C). This analysis showed that, similar to colchicine, the microtubules of Microtubin-1, Microtubin-2, and Microtubin-3-treated cells became destabilized in a drug dose-dependent manner (Figure 4C).

### Microtubins arrest cells in mitosis and activate the spindle assembly checkpoint

**Figure 5:**
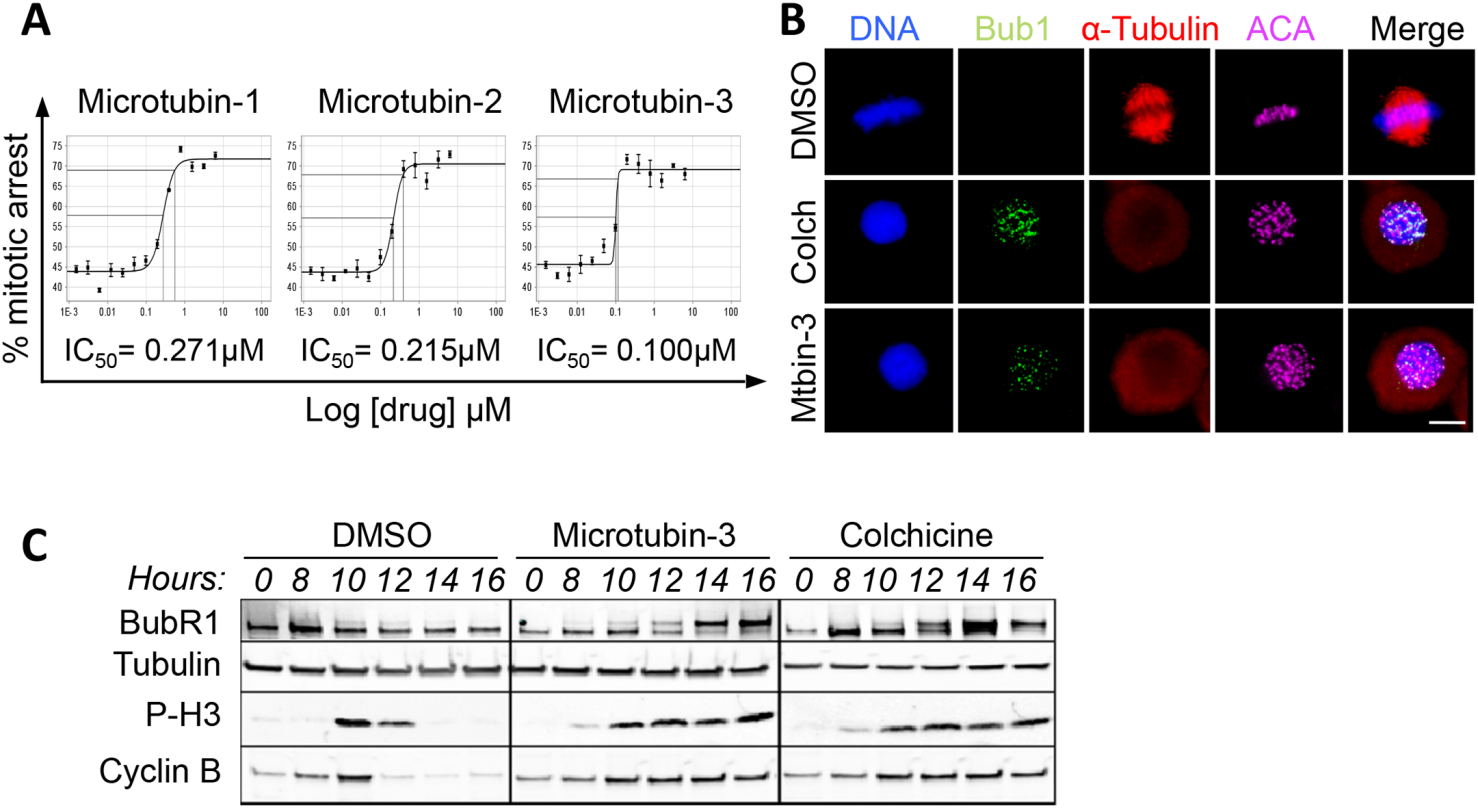
The Microtubins arrest cells in mitosis with an active spindle assembly checkpoint **(A)**, HeLa cells were treated with increasing concentrations of Microtubin-1, Microtubin-2, or Microtubin-3 for 20 hours and the drug response dose curves were used to measure the mitotic arrest IC_50s_ for each treatment. Graphs display % mitotic arrest on the y-axis and increasing concentrations of the indicated drugs on the x-axis. **(B)**, immunofluorescence microscopy of cells treated with DMSO, colchicine (100 nM), and Microtubin-3 (100 nM) showing that Microtubin-3-treated cells arrest in mitosis with an activate spindle assembly checkpoint (Bub1 remains at the centromere/kinetochore region) similar to colchicine treatment. Bar indicates 5μm. **(C)**, HeLa cells were arrested in G1/S, released into the cell cycle in the presence or absence of DMSO, colchicine (100 nM) or Microtubin-3 (100 nM) and protein extracts were immunoblotted at the indicated time points. Immunoblot analysis shows that Microtubin-3-treated cells arrest in mitosis (increased P-H3 signal) with an active spindle assembly checkpoint (BubR1 phosphorylation is present as a higher mobility band), similar to colchicine treatment. Experiment was performed three times. Shown are representative blots.

To further understand the Microtubin mechanism of action, we first verified that the most potent Microtubin-1 analogues Microtubin-2 and Microtubin-3 were also arresting cells in mitosis, by using the Vybrant DyeCycle Green cell cycle analysis assay described above. Indeed, Microtubin-2 and Microtubin-3-treated cells arrested in mitosis (72.6% and 72.1%, respectively) (Figure 5A). Additionally, the mitotic arrest IC_50_ for these compounds (Microtubin-1 = 271 nM, Microtubin-2 = 215 nM and Microtubin-3 = 100 nM) was similar to their cell viability IC_50_ (Figure 5A and Table 2). Next, we asked if the spindle assembly checkpoint (SAC) was activated in cells treated with the most potent compound Microtubin-3, as observed with other antimitotic treatments [30]. Microtubin-3-treated cells were fixed and co-stained for DNA (with the DNA dye Hoechst 33342), centromeres (anti-centromere antibodies, ACA) and the SAC component Bub1 (with anti-Bub1 antibodies), which remains associated with kinetochores when the SAC is active. Indeed, Bub1 remained localized to the centromeric/kinetochore region (co-localized with the ACA signal) in colchicine and Microtubin-3-treated cells, indicative of checkpoint activation (Figure 5B). To further verify that Microtubin-3 was activating the SAC, we synchronized HeLa cells in G1/S, released them into the cell cycle in the presence of DMSO, colchicine, or Microtubin-3 and cells were harvested every two hours upon entry into mitosis. Cells were then lysed and protein extracts were analyzed by immunoblotting. Consistent with the immunofluorescence microscopy data, this analysis showed that Microtubin-3-treated cells arrested in mitosis (increased P-H3 signal) with an active spindle assembly checkpoint (BubR1 remained phosphorylated), similar to colchicine-treated cells (Figure 5C).

### Live-cell analysis of microtubin-treated cells

**Figure 6:**
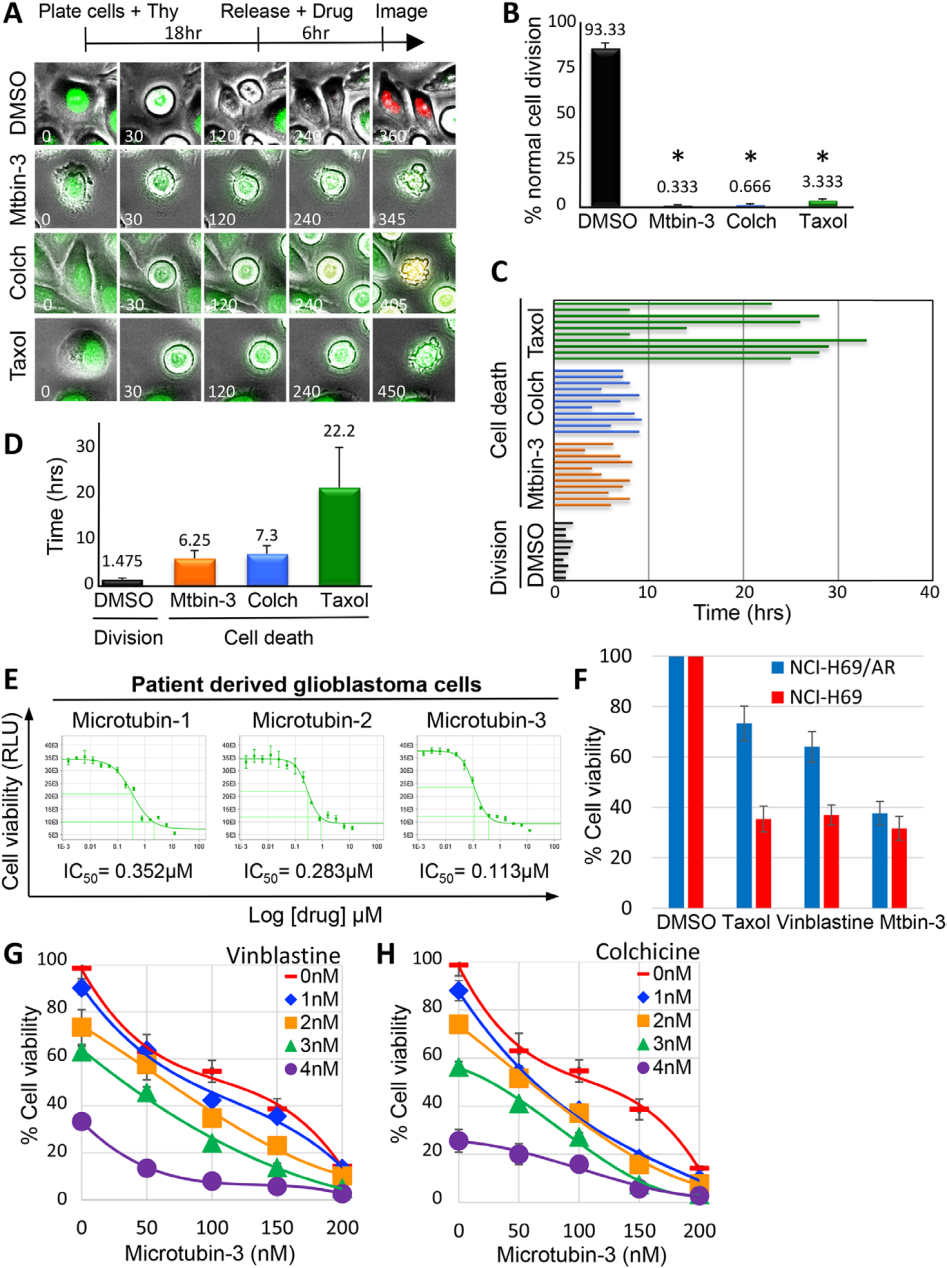
Live-cell analysis of Microtubin-induced cell death **(A)**, live-cell time-lapse microscopy of HeLa FUCCI cells treated with DMSO, colchicine (100 nM), Microtubin-3 (100 nM), or taxol (10 nM). Time is in minutes. See also Supplementary Videos 1-4. **(B)**, the percentage of cells undergoing normal cell division was quantified for DMSO, colchicine, Microtubin-3, or taxol-treated cells. Graph displays % normal cell divisions on the y-axis for the indicated drug treatments on the x-axis. Data represent the average ± SD of 3 independent experiments, with 20 cells counted for each. Asterisks denotes p-value <.0001. **(C)**, individual cells treated with indicated drugs were tracked over time using live-cell time-lapse microcopy and the length of time from mitotic entry to cell death was represented as a bar for each cell. **(D)**, the length of time from mitotic entry to cell death was quantified for DMSO, colchicine, Microtubin-3, or taxol-treated cells. Graph displays time (in hours) on the y-axis for the indicated drug treatments on the x-axis. Data represent the average ± SD of 3 independent experiments, with 10 cells counted for each. **(E)**, Microtubins are inhibitors of glioblastoma tumor cell proliferation. Patient-derived glioblastoma cells (HK-309) were treated with a fourteen point two-fold titration (1.5 nM to 12.5 μM) of Microtubin-1, Microtubin-2, or Microtubin-3 for 72 hours and their cell viability (CellTiter-Glo Assay) IC_50_ was determined. Graphs display cell viability on the y-axis (RLU indicates relative light units) and increasing concentrations of the indicated drugs on the x-axis. **(F)**, Microtubin-3 inhibits multi-drug resistant small cell lung carcinoma cells. Parental NCI-H69 and multi-drug resistant NCI-H69/AR (overexpress MRP1) small cell lung carcinoma cells were treated with Microtubin-3 (200 nM), taxol (20 nM), or vinblastine (2.5 nM) at the indicated concentrations for 72 hours. Cell viability was then determined using the CellTiter-Glo assay. Graph displays % cell viability on the y-axis and the indicated drug treatments on the x-axis for NCI-H69 (red bars) NCI-H69/AR (blue bars) cell lines. Error bars indicate standard deviations from 3 independent triplicate experiments. **(G-H)**, drug-drug interaction studies with Microtubin-3 and vinblastine (G) and Microtubin-3 and colchicine (H). HeLa cells were treated with increasing concentrations of Microtubin-3, vinblastine and colchicine alone or in combination for 48 hours and the drug response dose curves were used to measure the cell viability (CellTiter-Glo assay) for each treatment. Graphs display % cell viability on the y-axis and increasing concentrations of Microtubin-3 on the x-axis for each drug combination.

To further understand the cellular consequences of treating cancer cells with this new class of compounds, we coupled cell synchronizations with live-cell time-lapse immunofluorescence microscopy. HeLa FUCCI cells (fluorescent ubiquitination-based cell cycle indicator cell line [31]), which express a green fluorescent protein fused to human Geminin (mAG–hGeminin) from S phase through M phase and express a red fluorescent protein fused to human Cdt1 (mKO2-hCdt1) during G1 phase [31]) were synchronized in G1/S, released into the cell cycle, and treated with DMSO, colchicine (100 nM), Microtubin-3 (300 nM), or taxol (100 nM) for two hours prior to mitotic entry (Figure 6A). The effect of each treatment on cell division was assessed by capturing images at fifteen-minute intervals. Each capture consisted of ten 0.9 μm optical sections at 20X magnification, which were deconvolved and collapsed as maximum intensity projection images to give a detailed view of the cell (Figure 6A). The images were then processed into video format (Supplementary Videos 1-4 for DMSO, colchicine, Microtubin-3 and taxol respectively) and analyzed to determine the percentage of cells that divided normally and the total elapsed time between mitotic entry and cell death [32] (Figure 6B, 6C and 6D). The majority of control DMSO-treated cells transitioned through mitosis (green fluorescence) and into G1 (red fluorescence) normally (93.3% ± 3.1) (Figure 6A and 6B). In contrast, Microtubin-3, colchicine and taxol-treated cells arrested in mitosis and apoptosed with few cells dividing (Microtubin-3= 0.33% ± 0.5, p<.0001; colchicine= 0.66% ± 0.6, p<.0001; taxol= 3.3% ± 0.5, p<.0001) (Figure 6A and 6B). However, Microtubin-3 and colchicine-treated cells arrested for a shorter time-length than taxol prior to apoptosing (Microtubin-3= 6.25 ± 1.75 hours, colchicine= 7.3 ± 1.81 hours, taxol= 22.2 ± 8.97 hours, compared to DMSO= 1.48 ± 0.34 hours) (Figure 6C and 6D). These data indicated that Microtubin-3 was a cell cycle specific inhibitor, which arrested cells in mitosis, activated the SAC, and induced an apoptotic cell death with faster kinetics than taxol.

### The microtubins inhibit the proliferation of patient derived brain cancer cells and multi-drug resistant small cell lung carcinoma cells

To further evaluate the potential of the Microtubins to inhibit cancer cell proliferation, we analyzed their potency in primary cancer cells using patient derived glioblastoma cells (HK-309) and a fourteen point two-fold titration (1.5 nM to 12.5 μM) of Microtubin-1, Microtubin-2, or Microtubin-3 and determined their cell viability IC_50_ (Figure 6E). Interestingly, Microtubin-1, Microtubin-2 and Microtubin-3 showed great efficacy in these populations of brain cancer cells (cell viability IC_50_ for Microtubin-1 = 352 nM, Microtubin-2 = 283 nM, and Microtubin-3 = 113 nM) (Figure 6E). Additionally, we tested the ability of taxol, vinblastine and Microtubin-3 to reduce the viability of the small cell lung carcinoma cell line NCI-H69 and its derivative NCI-H69/AR that overexpresses the multidrug resistant protein (MRP1), which confers resistance to vinca alkaloids like vinblastine (and other chemotherapeutics like taxol, doxorubicin, mitoxantrone, and etoposide) compared to the parental NCI-H69 cell line [33]. Whereas, taxol and vinblastine showed a major decrease in their ability to kill NCI-H69/AR cells compared to NCI-H69 cells, Microtubin-3 was equally as effective at reducing the viability of both cell lines (Figure 6F). These results indicated that the Microtubins were not only effective against a cervical adenocarcinoma cell line, but also patient derived glioblastoma cells and multi-drug resistant small cell lung carcinoma cells. Finally, we explored the possibility that Microtubins could be combined with other microtubule targeting agents to enhance cancer cell killing. HeLa cells were treated with increasing concentrations of vinblastine or colchicine in combination with increasing concentrations of Microtubin-3 and cell viability was measured after 48 hours. Interestingly, Microtubin-3 enhanced vinblastine and colchicine mediated cell killing (Figure 6G and 6H). The data was then analyzed with the Chou-Talalay method where a drug combination index (CI) CI<1, =1, >1 indicates synergism, additive effect and antagonism, respectively [34]. This analysis determined that Microtubin-3 was synergizing with vinblastine and colchicine at the higher concentrations tested. For example, the Microtubin-3 (200 nM) and vinblastine (4 nM) interaction had a CI of .458 and the Microtubin-3 (200 nM) and colchicine (3 nM) interaction had a CI of .475. These data indicated that the Microtubins could potentially be used in combination with other antimitotics to enhance cancer cell killing.

## DISCUSSION

Since their discovery, microtubule targeting agents have become some of the most widely-used and effective anticancer agents with efficacy towards a broad range of cancers [4]. However, most of these agents are associated with several critical limitations to their production and use, including their derivation from natural sources, their large difficult to synthesize structures, their hydrophobic properties, their limited therapeutic window, their unwanted side effects like neutropenia, and their chemical properties that make them substrates of drug efflux pumps [4, 13]. This has prompted us and others to discover and develop novel synthetic small molecules that can address these issues associated with traditional microtubule targeting agents [35, 36]. Here, we have discovered and characterized the Microtubins, a novel class of “simple” small synthetic compounds that inhibit patient derived glioblastoma cell proliferation. The Microtubins function by targeting tubulin, inhibiting microtubule polymerization and formation of the mitotic spindle, arresting the cells in mitosis, activating the spindle assembly checkpoint, and triggering a fast apoptotic cell death. Our results suggest that simple chemical structures can be used to target microtubules and induce cancer cell death and should be considered in the development of novel anticancer drugs.

The testing of 26 Microtubin-1 analogues in cell culture structure-activity relationship studies indicated that the Microtubin phenyl ring is an important site that could be modified to further improve its activity. Ligand fluorination increases membrane accumulation of many CNS drugs by enhancing compound lipophilicity [37, 38]; thus, the improved activity of Microtubin-3 and Microtubin-2 over Microtubin-1 may be due to increased cellular permeability (Table 2). On the other hand, while the methoxy and methyl-sulfur groups also have strong lipophilic characters, the decrease in potency for Microtubin-4and Microtubin-5 in comparison to Microtubin-1, may be attributed to diminished solubility and membrane permeability from an increased molecular size [38] (Table 2).

Acquired resistance to microtubule targeting drugs whether by mutation of the drug binding site within tubulin, the overexpression of tubulin isoforms like βIII-Tubulin that is not a target of most microtubule targeting drugs, or the overexpression of multidrug resistance effector pumps is a growing concern in the treatment of cancer [4, 13]. Our data suggests that the Microtubins are likely binding outside of the two major sites targeted for depolymerization (the vinca and colchicine sites) and Microtubin-3 was effective in at least one multi-drug resistant cell line, thus it is possible that the Microtubins could be effective against cancers that acquire or are resistant to current microtubule poisons and should be explored further. Recently, Eribulin a Halichondrin B synthetic analogue with microtubule destabilizing activity has been effectively used in the clinics to treat multiple types of cancers including taxane resistant cancers [39]. X-ray crystal structure studies showed that Eribulin was not only binding to the vinca domain between two ɑ/β-tubulin dimers, but to the exposed β-tubulin subunits at the distal plus ends of microtubules [40]. We considered this unique mechanism of action for the Microtubins, however molecular modeling studies of Microtubin-3 at the Eribulin tubulin receptor site indicated that it was a very weak binder compared to Eribulin and was unlikely to be functioning through a Eribulin-like mechanism of action (Supplementary Figure 2). Although, we have shown that the Microtubins are effective *in vitro* in models of cervical adenocarcinomas, patient derived glioblastoma cells and multi-drug resistant small cell lung carcinoma cells, future studies should be aimed at defining the specific types of cancers that are the most responsive to Microtubins and the potential of combining Microtubins with other FDA approved oncology drugs to improve cancer cell killing.

## MATERIALS AND METHODS

### Materials

The following antibodies were used in this study: phospho-histone H3 (Ser10)-488 (p-H3-488, Cell Signaling), ɑ-tubulin (Serotec), Anti-Centromere-Antibodies (ACA, Cortex Biochem), Cyclin B (Santa Cruz), BubR1 (Abcam) and Bub1 (EMD Millipore). Affinipure FITC-, Cy3- and Cy5-conjugated secondary antibodies were from Jackson Immuno Research. The following reagents were used in this study: CellTiter-Glo Luminescent Cell Viability Assay (Promega), Caspase-Glo 3/7 (Promega), HTS-Tubulin polymerization assay kit (Cytoskeleton Inc), ProLong Gold anti-fade reagent (Invitrogen), Hoechst 33342 (Thermo Scientific) and Vybrant DyeCycle Green (Invitrogen). Microtubin-15-27 were purchased from MolPort at >95% purity by LCMS. Microtubin-1-14 were synthesized and purified to >95% purity.

### Cell culture

Adherent HeLa, HeLa-FUCCI and patient derived glioblastoma cells were grown in F12:DMEM 50:50 medium (Invitrogen) and NCI-H69 and NCI-H69/AR cells were grown in RPMI 1640 medium (Invitrogen) containing 10% FBS and 1% penicillin/streptomycin with 5% CO_2_ at 37°C. To obtain HeLa cells synchronized in mitosis, cycling cells were treated with 2 mM thymidine (Sigma-Aldrich) for eighteen hours, washed three times with PBS, and released into fresh media until they entered mitosis eight hours post-release. Patient-derived glioblastoma cells (HK-309) were collected and grown with approval from the UCLA Institutional Review Board. HK-309 was derived from a recurrent glioblastoma taken from a 55 year old male. The cells were initially propagated as cancer stem cell-containing spheres in serum-free medium containing basic fibroblast growth factor and epidermal growth factor (Preprotech) as described previously [41].

### Cell cycle analysis

For cell cycle analysis, HeLa cells were plated in 384 well plates (1500 cells/well) and treated with 10 μM drugs for 20 hours. Cells were then fixed and stained with 5 μM Vybrant DyeCycle Green (Invitrogen) for 3 hours at 37°C and plates were scanned with an Acumen ^e^X3 (TTP Labtech) fluorescence microplate cytometer using its 488 nm laser and a cell cycle histogram profile was generated for each drug treatment using the CDD (Collaborative Drug Discovery) software.

### Analogue design

To diversify the core scaffold of Microtubin-1, we used Microtubin-1 as a query to search the ZINC database (http://zinc.docking.org/) for compounds with greater than 80% chemical similarity. 397 analogues were retrieved from the database and subjected to a drug-like filter based on Lipinski’s rule of five, including less than five hydrogen bond donors, a molecular weight less than 500 Da, a logP less than 5 and less than 10 hydrogen bond acceptors [29]. From this, we selected 13 compounds for further analysis. Alternatively, 38 phenyl derivatives of Microtubin-1 were designed according to the Topliss scheme for aromatic ring optimization and subjected to the same drug-like filter [28]. Finally, 13 compounds from each category (a total of 26) were acquired or synthesized based on availability, ease of synthesis and functional diversity. See Supplementary Material for information on the synthesis of Microtubin-1-14.

### Compound IC_50s_

The cell viability IC_50_ of each compound was determined using Promega CellTiter-Glo Luminescent Cell Viability Assay kit by measuring the total ATP levels to quantify the number of metabolically active cells upon drug treatment as described in [42]. Briefly, the compounds were suspended in DMSO at 10 mM and diluted in 384 plates (20 μl/well in DMSO) in triplicate by a 14-point titration (12 nM to 100 μM). 50 μl of HeLa cells or patient derived glioblastoma cells (HK-309) (2000 cells/well) were then treated with the prepared dilutions of the drugs (0.5 μl) and incubated at 37°C and 5% CO_2_. 72 hours later 50 μl of CellTiter-Glo reagent was added to each well followed by 2 minutes shaking and a 10-minute incubation to lyse the cells. The relative luminescent intensity units (RLU) of each well was measured using a Tecan M1000 microplate reader (Tecan Group Ltd.) with its green filter and 1 second integration time. Similarly, to measure caspase activity in response to 24 hour compound treatment we used the Caspase-Glo 3/7 assay from Promega as described above for the CellTiter-Glo assay.

### Microtubule polymerization assays

Tubulin polymerization assays were conducted using the HTS-Tubulin polymerization assay kit from Cytoskeleton Inc, as described previously [26]. The reactions were carried out according to the manufacturer instructions (Cytoskeleton, BK011P) in the presence of DMSO or 15 μM Microtubins, colchicine or taxol. 1.5 ul of 10X strength Microtubin, 20 μl of tubulin solution and Triton X-100 at a final concentration of 0.01% were added to each well in a 384 well plate. The reactions were assembled on ice to prevent tubulin pre-polymerization. Tubulin polymerization was measured by the increase in fluorescence emission at 420 nm using a Tecan M1000 microplate reader (Tecan Group Ltd.). Fluorescence increased as polymerization occurred, due to the incorporation of 4’,6-diamidino-2-phenylindole. Fluorescence was monitored every minute for 70 minutes at 37**°**C.

### Competitive mass spectrometry assay

Competitive mass spectrometry assays were performed as described in [25, 26]. Briefly, colchicine or vinblastine (1.2 μM) were incubated with 1.0 mg/mL porcine tubulin in PIPES buffer (80 mM PIPES, 2.0 mM MgCl2, 0.5 mM EGTA, pH 6.9) at 37°C for one-hour in the absence of GTP. The indicated compounds (100 μM) were added to compete with the binding of colchicine or vinblastine to tubulin. After a one-hour incubation, the unbound ligands were separated from tubulin using a micro-concentrator (Microcon) with a 30kDa molecular weight cut-off. Samples were collected by centrifugation at 14,000 g for 30 minutes. 50 μl of filtrate was diluted with 150 μl of acetonitrile/H_2_O (1/2) containing 200 nM internal standard. A 10 μl eluate sample was analyzed by high performance liquid chromatography tandem mass spectrometry (LC-MS/MS) as described in [25] and the ability of the compounds of interest to inhibit the binding of colchicine or vinblastine to tubulin was expressed as a percentage of control binding in the absence of any competitor. Each experiment was performed in triplicate.

### Drug-drug interaction studies

HeLa cells were treated with increasing concentrations of Microtubin-3, vinblastine and colchicine alone or in combination for 48 hours and cell viability was measured using the CellTiter-Glo assay (Promega), which measures total ATP levels. Plates were read with a Tecan M1000 micro-plate reader (Tecan Group Ltd.) at 540nm. The average readout from the control DMSO-treated cells was used to calculate the average % cell viability of compound-treated cells. The data was analyzed with the Chou-Talalay method where the drug combination index (CI) CI<1, =1, >1 indicates synergism, additive effect and antagonism, respectively with the CompuSyn software (ComboSyn Inc.) [34].

### Fixed-cell microscopy

Immunofluorescence microscopy was carried out essentially as described previously [43]. Briefly, HeLa cells were arrested with 2mM thymidine for eighteen hours, washed three times with PBS, and released into fresh media. Nine hours later, cells were fixed with 4% paraformaldehyde, permeabilized with 0.2% Triton X-100/PBS and stained with 0.5 μg/ml Hoechst 33342, rat anti-ɑ-tubulin (Serotec) and other indicated antibodies. Slides were mounted with ProLong Gold anti-fade reagent (Invitrogen), images were captured with a Leica DMI6000 microscope (Leica-Microsystems) at 63X magnification with ten Z-stacks, one every 0.5 μm., and Z-stacks were compressed and displayed as projection images.

### Live-cell time-lapse microscopy

Live-cell time-lapse microscopy was carried out essentially as described previously [21]. HeLa-FUCCI cells were arrested with 2mM thymidine for eighteen hours, washed three times with PBS, and released into fresh media. Six hours post-release, cells were treated with indicated small molecules and imaged live at 20X magnification with ten Z-stacks, one every 1 μm, at fifteen-minute intervals. Images were captured with a Leica DMI6000 microscope (Leica Microsystems), processed using LSF software and converted to AVI videos. Each frame represents a fifteen-minute interval. Data quantitation represents the average ± SD (standard deviation) of 3 independent experiments, with 20 cells counted for each.

### Molecular modeling

The Eribulin-tubulin co-crystal structure was retrieved from the PDB database (PDB ID: 5JH7). The docking of Microtubin-3 to the Eribulin binding site was performed using the Molecular Operating Environment (MOE) program (version 2009, Chemical Computing Group) as described previously [26, 44]. To prepare the receptor for docking, the solvent and ions were computationally removed followed by protonation and tether minimization using the LigPrep protocol. Next, the Microtubin-3 and Eribulin were docked into the Eribulin site points using the triangle matching algorithm. The docked poses were first scored using the London dG scoring function, which estimated the free energy of binding from a given pose, followed by force field refinement and London dG rescoring. The top scoring docked poses of each molecule within the Eribulin site were retained.

## SUPPLEMENTARY MATERIALS FIGURES, TABLES AND VIDEOS

















## Abbreviations

VCR: vincristine
Podo: podophyllotoxin
SAC: spindle assembly checkpoint
SAR: structure-activity relationship
IC_50_: half maximal inhibitory concentration

## References

[1] Manchado E, Guillamot M, Malumbres M (2012). Killing cells by targeting mitosis. Cell Death Differ.

[2] Lansing TJ, McConnell RT, Duckett DR, Spehar GM, Knick VB, Hassler DF, Noro N, Furuta M, Emmitte KA, Gilmer TM, Mook RA, Cheung M (2007). *In vitro* biological activity of a novel small-molecule inhibitor of polo-like kinase 1. Mol Cancer Ther.

[3] Kapoor TM, Mayer TU, Coughlin ML, Mitchison TJ (2000). Probing spindle assembly mechanisms with monastrol, a small molecule inhibitor of the mitotic kinesin, Eg5. J Cell Biol.

[4] Dumontet C, Jordan MA (2010). Microtubule-binding agents: a dynamic field of cancer therapeutics. Nat Rev Drug Discov.

[5] Gascoigne KE, Taylor SS (2008). Cancer cells display profound intra- and interline variation following prolonged exposure to antimitotic drugs. Cancer Cell.

[6] Shi J, Orth JD, Mitchison T (2008). Cell type variation in responses to antimitotic drugs that target microtubules and kinesin-5. Cancer Res.

[7] Matson DR, Stukenberg PT (2011). Spindle poisons and cell fate: a tale of two pathways. Mol Interv.

[8] Wertz IE, Kusam S, Lam C, Okamoto T, Sandoval W, Anderson DJ, Helgason E, Ernst JA, Eby M, Liu J, Belmont LD, Kaminker JS, O’Rourke KM (2011). Sensitivity to antitubulin chemotherapeutics is regulated by MCL1 and FBW7. Nature.

[9] Colin DJ, Hain KO, Allan LA, Clarke PR (2015). Cellular responses to a prolonged delay in mitosis are determined by a DNA damage response controlled by Bcl-2 family proteins. Open Biol.

[10] Orth JD, Loewer A, Lahav G, Mitchison TJ (2012). Prolonged mitotic arrest triggers partial activation of apoptosis, resulting in DNA damage and p53 induction. Mol Biol Cell.

[11] Bates D, Eastman A (2017). Microtubule destabilising agents: far more than just antimitotic anticancer drugs. Br J Clin Pharmacol.

[12] Komlodi-Pasztor E, Sackett D, Wilkerson J, Fojo T (2011). Mitosis is not a key target of microtubule agents in patient tumors. Nat Rev Clin Oncol.

[13] Canta A, Chiorazzi A, Cavaletti G (2009). Tubulin: a target for antineoplastic drugs into the cancer cells but also in the peripheral nervous system. Curr Med Chem.

[14] Carlson K, Ocean AJ (2011). Peripheral neuropathy with microtubule-targeting agents: occurrence and management approach. Clin Breast Cancer.

[15] Rivera E, Gomez H (2010). Chemotherapy resistance in metastatic breast cancer: the evolving role of ixabepilone. Breast Cancer Res.

[16] Senese S, Lo YC, Huang D, Zangle TA, Gholkar AA, Robert L, Homet B, Ribas A, Summers MK, Teitell MA, Damoiseaux R, Torres JZ (2014). Chemical dissection of the cell cycle: probes for cell biology and anti-cancer drug development. Cell Death Dis.

[17] Jeon JY, An JH, Kim SU, Park HG, Lee MA (2008). Migration of human neural stem cells toward an intracranial glioma. Exp Mol Med.

[18] Veber DF, Johnson SR, Cheng HY, Smith BR, Ward KW, Kopple KD (2002). Molecular properties that influence the oral bioavailability of drug candidates. J Med Chem.

[19] Lipinski CA (2000). Drug-like properties and the causes of poor solubility and poor permeability. J Pharmacol Toxicol Methods.

[20] Clark DE (1999). Rapid calculation of polar molecular surface area and its application to the prediction of transport phenomena. 2. Prediction of blood-brain barrier penetration. J Pharm Sci.

[21] Torres JZ, Summers MK, Peterson D, Brauer MJ, Lee J, Senese S, Gholkar AA, Lo YC, Lei X, Jung K, Anderson DC, Davis DP, Belmont L, Jackson PK (2011). The STARD9/Kif16a kinesin associates with mitotic microtubules and regulates spindle pole assembly. Cell.

[22] Hendzel MJ, Wei Y, Mancini MA, Van Hooser A, Ranalli T, Brinkley BR, Bazett-Jones DP, Allis CD (1997). Mitosis-specific phosphorylation of histone H3 initiates primarily within pericentromeric heterochromatin during G2 and spreads in an ordered fashion coincident with mitotic chromosome condensation. Chromosoma.

[23] Fojo AT (2008). The role of microtubules in cell biology, neurobiology, and oncology.

[24] Botta M, Forli S, Magnani M, Manetti F (2009). Molecular modeling approaches to study the binding mode on tubulin of microtubule destabilizing and stabilizing agents. Top Curr Chem.

[25] Li CM, Lu Y, Ahn S, Narayanan R, Miller DD, Dalton JT (2010). Competitive mass spectrometry binding assay for characterization of three binding sites of tubulin. J Mass Spectrom.

[26] Lo YC, Senese S, Li CM, Hu Q, Huang Y, Damoiseaux R, Torres JZ (2015). Large-scale chemical similarity networks for target profiling of compounds identified in cell-based chemical screens. PLOS Comput Biol.

[27] Irwin JJ, Shoichet BK (2005). ZINC—a free database of commercially available compounds for virtual screening. J Chem Inf Model.

[28] Jones SG, Singh PK, Jones MM (1988). Use of the Topliss scheme for the design of more effective chelating agents for cadmium decorporation. Chem Res Toxicol.

[29] Lipinski CA, Lombardo F, Dominy BW, Feeney PJ (2001). Experimental and computational approaches to estimate solubility and permeability in drug discovery and development settings. Adv Drug Deliv Rev.

[30] Chan KS, Koh CG, Li HY (2012). Mitosis-targeted anti-cancer therapies: where they stand. Cell Death Dis.

[31] Sakaue-Sawano A, Kurokawa H, Morimura T, Hanyu A, Hama H, Osawa H, Kashiwagi S, Fukami K, Miyata T, Miyoshi H, Imamura T, Ogawa M, Masai H, Miyawaki A (2008). Visualizing spatiotemporal dynamics of multicellular cell-cycle progression. Cell.

[32] Hutchins JR, Toyoda Y, Hegemann B, Poser I, Hériché JK, Sykora MM, Augsburg M, Hudecz O, Buschhorn BA, Bulkescher J, Conrad C, Comartin D, Schleiffer A (2010). Systematic analysis of human protein complexes identifies chromosome segregation proteins. Science.

[33] Mirski SE, Gerlach JH, Cole SP (1987). Multidrug resistance in a human small cell lung cancer cell line selected in adriamycin. Cancer Res.

[34] Chou TC, Talalay P (1984). Quantitative analysis of dose-effect relationships: the combined effects of multiple drugs or enzyme inhibitors. Adv Enzyme Regul.

[35] Kavallaris M, Verrills NM, Hill BT (2001). Anticancer therapy with novel tubulin-interacting drugs. Drug Resist Updat.

[36] McNamara DE, Senese S, Yeates TO, Torres JZ (2015). Structures of potent anticancer compounds bound to tubulin. Protein Sci.

[37] Katritzky AR, Dobchev DA, Fara DC, Hür E, Tämm K, Kurunczi L, Karelson M, Varnek A, Solov’ev VP (2006). Skin permeation rate as a function of chemical structure. J Med Chem.

[38] Kerns EH, Di L (2008). Drug-like properties : concepts, structure design and methods : from ADME to toxicity optimization.

[39] Swami U, Shah U, Goel S (2015). Eribulin in Cancer Treatment. Mar Drugs.

[40] Doodhi H, Prota AE, Rodríguez-García R, Xiao H, Custar DW, Bargsten K, Katrukha EA, Hilbert M, Hua S, Jiang K, Grigoriev I, Yang CP, Cox D (2016). Termination of Protofilament Elongation by Eribulin Induces Lattice Defects that Promote Microtubule Catastrophes. Curr Biol.

[41] Visnyei K, Onodera H, Damoiseaux R, Saigusa K, Petrosyan S, De Vries D, Ferrari D, Saxe J, Panosyan EH, Masterman-Smith M, Mottahedeh J, Bradley KA, Huang J (2011). A molecular screening approach to identify and characterize inhibitors of glioblastoma stem cells. Mol Cancer Ther.

[42] Crouch SP, Kozlowski R, Slater KJ, Fletcher J (1993). The use of ATP bioluminescence as a measure of cell proliferation and cytotoxicity. J Immunol Methods.

[43] Torres JZ, Ban KH, Jackson PK (2010). A specific form of phospho protein phosphatase 2 regulates anaphase-promoting complex/cyclosome association with spindle poles. Mol Biol Cell.

[44] Lo YC, Senese S, Damoiseaux R, Torres JZ (2016). 3D Chemical Similarity Networks for Structure-Based Target Prediction and Scaffold Hopping. ACS Chem Biol.

