# Supplementary material for: Microtubins: a novel class of small synthetic microtubule targeting drugs that inhibit cancer cell proliferation
Source: Oncotarget. 2017 Oct 19;8(61):104007–21. doi: 10.18632/oncotarget.21945 (PMC5732783; doi:10.18632/oncotarget.21945)
Supplement: Supplementary file 1 [file oncotarget-08-104007-s001.pdf]

Microtubins: a novel class of small synthetic microtubule targeting drugs that inhibit cancer cell proliferation

SUPPLEMENTARY METHODS

Synthesis and characterization of microtubuin-1-14

All reagents and solvents were purchased from commercial suppliers and used without further purification unless otherwise stated. The reactions were monitored by thin layer chromatography (TLC) on precoated silica gel F254 plates (Sigma􀀀Aldrich) with a UV indicator using chloroform:methanol (9.5:0.5 v/v). Yields were of purified product and were not optimized. The purity of the newly synthesized compounds was determined by LCMS analysis. The proton nuclear resonance (1H NMR) spectra were performed on a Varian GEMINI 2000 NMR spectrometer system with working frequency 400MHz. Chemical shifts δ are given in ppm, and the following abbreviations are used: singlet (s), doublet (d), triplet (t), quartet (q), multiplet (m), and broad singlet (br s). All LCMS data were gathered on an Agilent 1100 LC system. The compound solution was injected into the ionization source (APCI) operating positive and negative modes with a mobile phase acetonitrile/water/formic acid (50:50:0.1% v/v) at 1.0 mL/min. The instrument was externally calibrated for the mass range m/z 100 to m/z 650.

General Procedure for the Preparation of Ethyl-2-amino-4,5,6,7-tetrahydrobenzo[b] thiophene-3-carboxylate-Morpholine (20.4 g, 0.234 mol) was added dropwise to a mixture of cyclohexanone (10.0 g, 0.102 mol), ethyl cyanoacetate (11.53 g, 0.102 mol), and sulfur (2.9 g, 0.094 mol) in 30mL of ethanol at ambient temperature in 20 min. The reaction was exothermic, so the temperature increased to 50°C, and a clear solution was obtained. It was filtered from solids and cooled to 0–5°C. After stirring for 1h the product was filtered off, washed with chilled ethanol (15 mL) and sucked dry. The obtained cake was dried in an oven at 50°C for 3h to obtain 14g of the title product. Yield: 66.0%. 1H NMR (ppm): 1.23 (3H, t), 1.61–1.72 (4H, m), 2.38–2.41 (2H, m), 2.56–2.61 (2H, m), 4.13 (2H, d), 7.21 (2H, br.s).

General Procedure for the Preparation of 2-(Chloromethyl)-5,6,7,8-tetrahydrobenzo [4, 5] thieno[2,3-d]pyrimidin-4(3H)-one-Ethyl-2-amino-4,5,6,7-tetrahydrobenzothiophene-3-carboxylate (11.25 g, 0.05mol) and chloroacetonitrile (5.0 g, 0.06 mol) were dissolved 1,4-dioxane. The obtained solution was heated to 50°C, and passed with dry hydrogen chloride gas for 3h until the starting material disappeared. Then the solvent was removed under vacuum. The residue was triturated with hexane, and a precipitate was formed as a fine powder. The product was filtered, washed with hexane, and air dried to obtain 8.1 g of the compound. Yield: 63.0%. 1H NMR (ppm): 1.71–1.82 (4H, m), 2.74 (2H, d.tr), 2.87 (2H, d.tr), 4.53 (2H, s), 12.5 (1H, br.s).

General Procedure for the Preparation of ((4-Oxo-3,4,5,6,7,8-hexahydrobenzo [4, 5] thieno[2,3-d]pyrimidin-2-yl)methyl) triphenylphosphonium chloride-0,01 mol of the compound II and 0,01 mol of thriphenylphosphine were boiled in 50 mL of toluene for 10h. The resulting residue was filtered off, washed with toluene, air dried and used for condensation with aldehydes without further purification. Yield: 75%. 1H NMR (ppm): 1.60–1.80 (4H, m), 12.90 (1H, br.s), 2.65 (2H, d.tr), 2.77 (2H, d.tr), 8.60 (2H, d), 8.70–8.80 (6H, m), 8.80–8.90 (9H, m).

General Procedure for the Preparation of Microtubin-1 and its Analogs Microtubin-2 through Microtubin-14- A 10% solution of Na_2CO_3 (0,8 mL) was added dropwise to a solution of thriphenylphosphonium chloride III (0,001 mol) and corresponding aldehyde (0,0005 mol) in methanol. Reaction mixture was stirred for 10 min at RT. The resulting yellow precipitate was filtered off, washed with water and methanol. The remaining residue was boiled in ethanol for 2h, filtered hot, washed with alcohol and dried to give the final compound as a solid.

(E)-2-styryl-5,6,7,8-tetrahydrobenzo [4, 5] thieno[2,3-d]pyrimidin-4(3H)-one (Microtubin-1). Yield: 46%. Purity 95% by LCMS. 1H NMR (ppm): 1.70–1.82 (4H, m), 2.75 (2H, d.t), 2.59 (2H, d.t), 6.98 (1H, d), 7.39–7.51 (3H, m), 7.68–7.81 (2H, m), 7.98 (1H, d), 12.97 (1H, br.s).

*(E)-2-(2,4-difluorostyryl)-5,6,7,8-tetrahydrobenzo [4, 5]thieno[2,3-d]pyrimidin-4(3H)-one (*Microtubin-2*).* Yield: 40%. Purity 95% by LCMS. 1H NMR (DMSO-d6, 400 MHz): δ 1.76-1.86 (4H, m), 2.75 (2H, d.t), 2.88 (2H, d.t), 7.03 (1H, d), 7.18-7.22 (1H, m), 7.34-7.41 (1H, m), 7.76-7.82 (1H, m), 7.86 (1H, d), 12.42 (1H, br.s).

*(E)-2-(2,5-difluorostyryl)-5,6,7,8-tetrahydrobenzo [4, 5]thieno[2,3-d]pyrimidin-4(3H)-one (*Microtubin-3*).* Yield: 42%. Purity 98% by LCMS. 1H NMR (DMSO-d6, 400 MHz): δ 1.76-1.86 (4H, m), 2.75 (2H, d.t), 2.89 (2H, d.t), 7.12 (1H, d), 7.27-7.40 (2H, m), 7.59-7.62 (1H, m), 7.86 (1H, d), 12.45 (1H, br.s).

*(E)-2-(4-fluorostyryl)-5,6,7,8-tetrahydrobenzo [4, 5] thieno[2,3-d]pyrimidin-4(3H)-one (*Microtubin-4*).* Yield: 33%. Purity 97% by LCMS. 1H NMR (DMSO-d6, 400 MHz): δ 1.76-1.86 (4H, m), 2.75 (2H, d.t), 2.89 (2H, d.t),

6.92 (1H, d), 7.24-7.32 (2H, m), 7.65-7.73 (2H, m), 7.87 (1H, d), 12.30 (1H, br.s).

(E)-2-(3-methoxystyryl)-5,6,7,8-tetrahydrobenzo [4, 5]thieno[2,3-d]pyrimidin-4(3H)-one (Microtubin-5). Yield: 36%. Purity 98% by LCMS. 1H NMR (DMSO-d6, 400 MHz): δ 1.75-1.88 (4H, m), 2.75 (2H, d.t), 2.88 (2H, d.t), 3.80 (3H, s) 6.98 (1H, d), 6.99 (1H, dd), 7.20 (1H, d), 7.21 (1H, d), 7.36 (1H, t), 7.84 (1H, d), 12.30 (1H, br.s).

*(E)-2-(4-methylthiostyryl)-5,6,7,8-tetrahydrobenzo [4, 5]thieno[2,3-d]pyrimidin-4(3H)-one (*Microtubin-6*).* Yield: 39%. Purity 95% by LCMS. 1H NMR (DMSO-d6, 400 MHz): δ 1.76-1.86 (4H, m), 2.52 (3H, s), 2.76 (2H, d.t), 2.91 (2H, d.t), 6.92 (1H, d), 7.32 (2H, d), 7.55 (2H, d), 7.84 (1H, d), 11.95 (1H, br.s).

*(E)-2-(4-ethoxy-3-methoxystyryl)-5,6,7,8-tetrahydrobenzo [4, 5]thieno[2,3-d]pyrimidin-4(3H)-one (*Microtubin-7*).* Yield: 46%. Purity 96% by LCMS. 1H NMR (DMSO-d6, 400 MHz): δ 1.34 (3H, t), 1.76-1.86 (4H, m), 2.75 (2H, d.t), 2.89 (2H, d.t), 3.82 (3H, s), 4.06 (2H, q), 6.86 (1H, d), 7.00 (1H, d), 7.16 (1H,dd), 7.24 (1H, d), 7.82 (1H, d), 12.20 (1H, br.s).

*(E)-2-(3-methylstyryl)-5,6,7,8-tetrahydrobenzo [4, 5]thieno[2,3-d]pyrimidin-4(3H)-one (*Microtubin-8*).* Yield: 56%. Purity 95% by LCMS. 1H NMR (DMSO-d6, 400 MHz): δ 1.76-1.86 (4H, m), 2.36 (3H, s), 2.76 (2H, d.t), 2.91 (2H, d.t), 6.96 (1H, d), 7.22 (1H, dd), 7.33 (1H, t), 7.42 (1H, d), 7.43 (1H, d), 7.83 (1H, d), 12.15 (1H, br.s).

*(E)-2-(4-methoxystyryl)-5,6,7,8-tetrahydrobenzo [4, 5]thieno[2,3-d]pyrimidin-4(3H)-one (*Microtubin-9*).* Yield: 55%. Purity 95% by LCMS. 1H NMR (DMSO-d6, 400 MHz): δ 1.75-1.88 (4H, m), 2.74 (2H, d.t), 2.88 (2H, d.t), 3.80 (3H, s) 6.83 (1H, d), 7.01 (2H, d), 7.58 (2H, d), 7.83 (1H, d), 12.24 (1H, br.s).

*(E)-2-(4-bromostyryl)-5,6,7,8-tetrahydrobenzo [4, 5]thieno[2,3-d]pyrimidin-4(3H)-one (*Microtubin-10*).* Yield: 39%. Purity 96% by LCMS. 1H NMR (DMSO-d6, 400 MHz): δ 1.75-1.88 (4H, m), 2.75 (2H, d.t), 2.88 (2H, d.t), 6.98 (1H, d), 7.58 (2H, d), 7.64 (2H, d), 7.83 (1H, d), 12.34 (1H, br.s).

*(E)-2-(3,4-difluorostyryl)-5,6,7,8-tetrahydrobenzo [4, 5]thieno[2,3-d]pyrimidin-4(3H)-one (*Microtubin-11*).* Yield: 53%. Purity 95% by LCMS. 1H NMR (DMSO-d6, 400 MHz): δ 1.76-1.90 (4H, m), 2.76 (2H, d.t), 2.90 (2H, d.t), 6.95 (1H, d), 7.44-7.52 (2H, m), 7.64-7.71 (1H, m), 7.82 (1H, d), 12.10 (1H, br.s).

*(E)-2-(3,4-dimethoxystyryl)-5,6,7,8-tetrahydrobenzo [4, 5]thieno[2,3-d]pyrimidin-4(3H)-one (*Microtubin-12*).* Yield: 30%. Purity 95% by LCMS. 1H NMR (DMSO-d6, 400 MHz): δ 1.76-1.86 (4H, m), 2.75 (2H, d.t), 2.89 (2H, d.t), 3.80 (3H, s), 3.81 (3H, s), 6.86 (1H, d), 7.02 (1H, d), 7.19 (1H, dd), 7.24 (1H, d), 7.82 (1H, d), 12.45 (1H, br.s).

*(E)-2-(4-ethoxystyryl)-5,6,7,8-tetrahydrobenzo [4, 5]thieno[2,3-d]pyrimidin-4(3H)-one (*Microtubin-13*).* Yield: 52%. Purity 95% by LCMS. 1H NMR (DMSO-d6, 400 MHz): δ 1.36 (3H, t), 1.76-1.86 (4H, m), 2.75 (2H, d.t), 2.89 (2H, d.t), 4.11 (2H, q), 6.82 (1H, d), 6.99 (2H, d), 7.55 (2H, d), 7.81 (1H, d), 11.90 (1H, br.s).

*(E)-2-(4-methylstyryl)-5,6,7,8-tetrahydrobenzo [4, 5]thieno[2,3-d]pyrimidin-4(3H)-one (*Microtubin-14*).* Yield: 41%. Purity 95% by LCMS. 1H NMR (DMSO-d6, 400 MHz): δ 1.76-1.86 (4H, m), 2.35 (3H, s), 2.76 (2H, d.t), 2.91 (2H, d.t), 6.92 (1H, d), 7.25 (2H, d), 7.52 (2H, d), 7.83 (1H, d), 12.05 (1H, br.s).

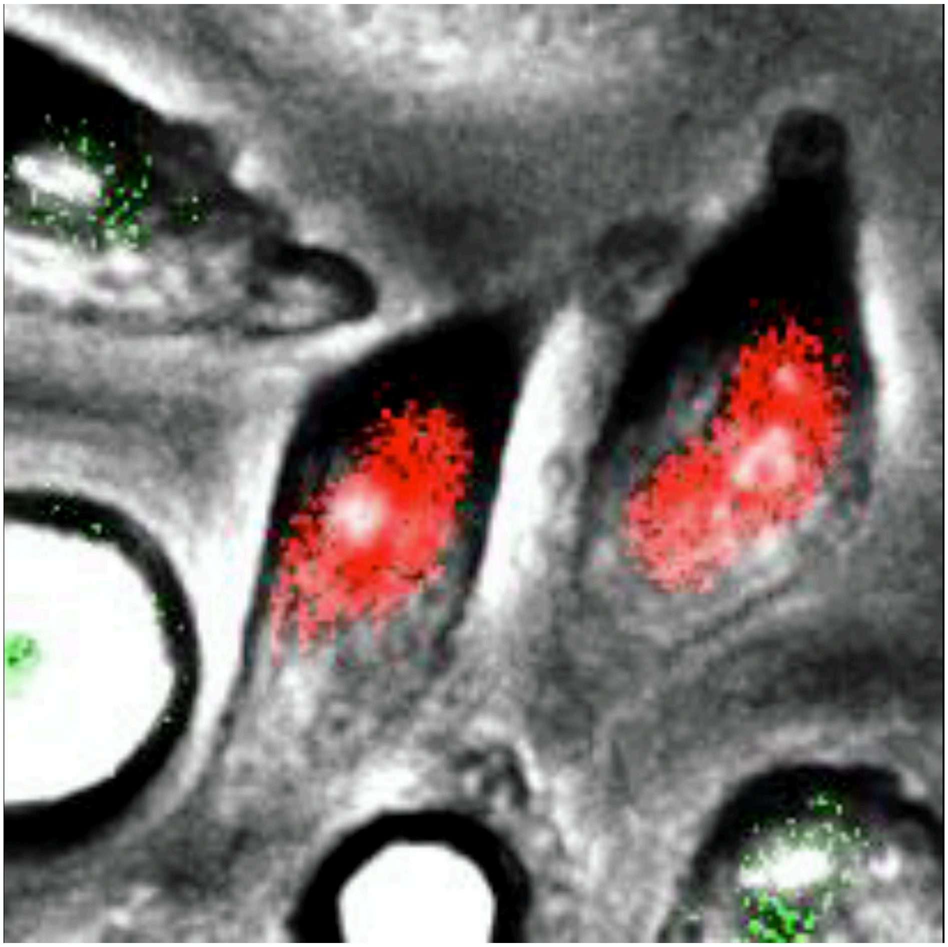

Supplementary Video 1: DMSO-treated control cell undergoing mitosis. Live time-lapse microscopy of control DMSO-treated cells. HeLa-FUCCI cells were arrested with Thymidine for 18 hours, washed, released into fresh media and DMSO was added 6 hours post release. Images from 3 channels (phase contrast, FITC and Cy3) were captured every 15 minutes at 20X magnification with a Leica DMI6000 microscope and processed using Leica deconvolution software (Leica Microsystems) and converted to an AVI movie file. Each frame represents a fifteen-minute interval.

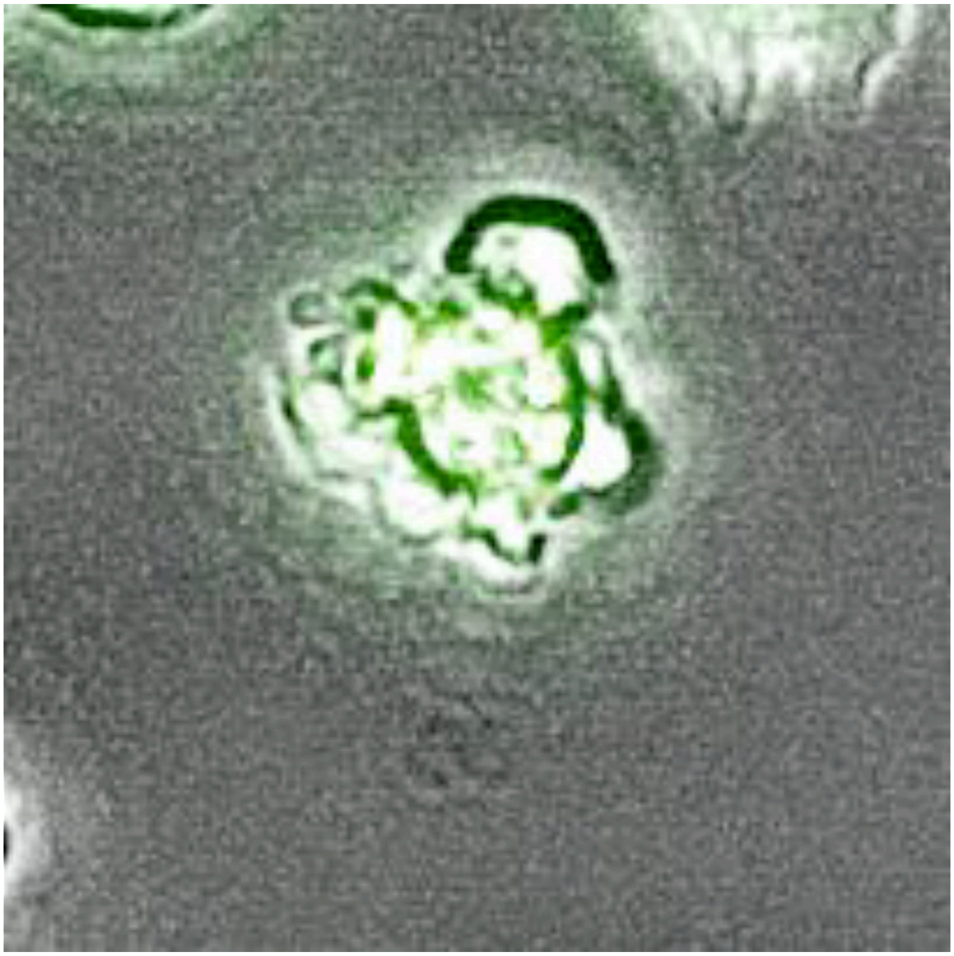

Supplementary Video 2: Microtubin-3-treated cell undergoing mitosis. HeLa-FUCCI cells were synchronized, treated with Microtubin-3, and imaged by live time-lapse microscopy as described for Video S1.

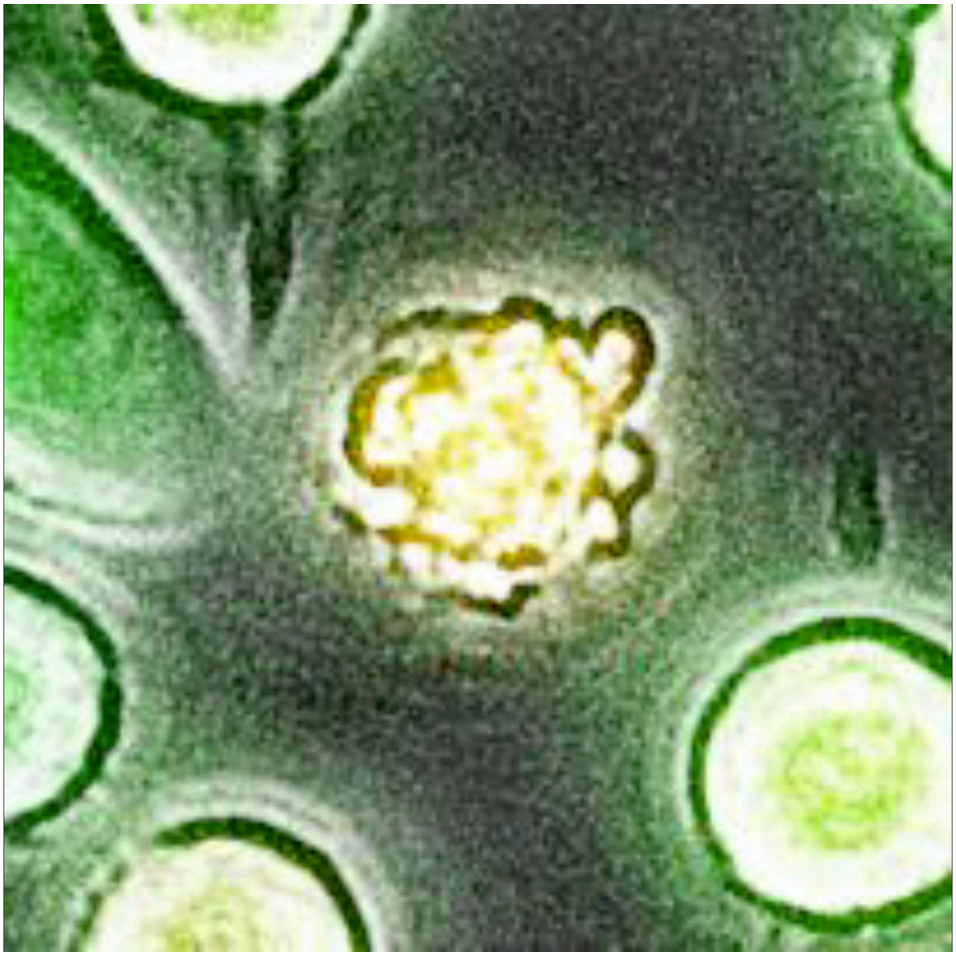

Supplementary Video 3: Colchicine-treated cell undergoing mitosis. HeLa-FUCCI cells were synchronized, treated with colchicine, and imaged by live time-lapse microscopy as described for Video S1.

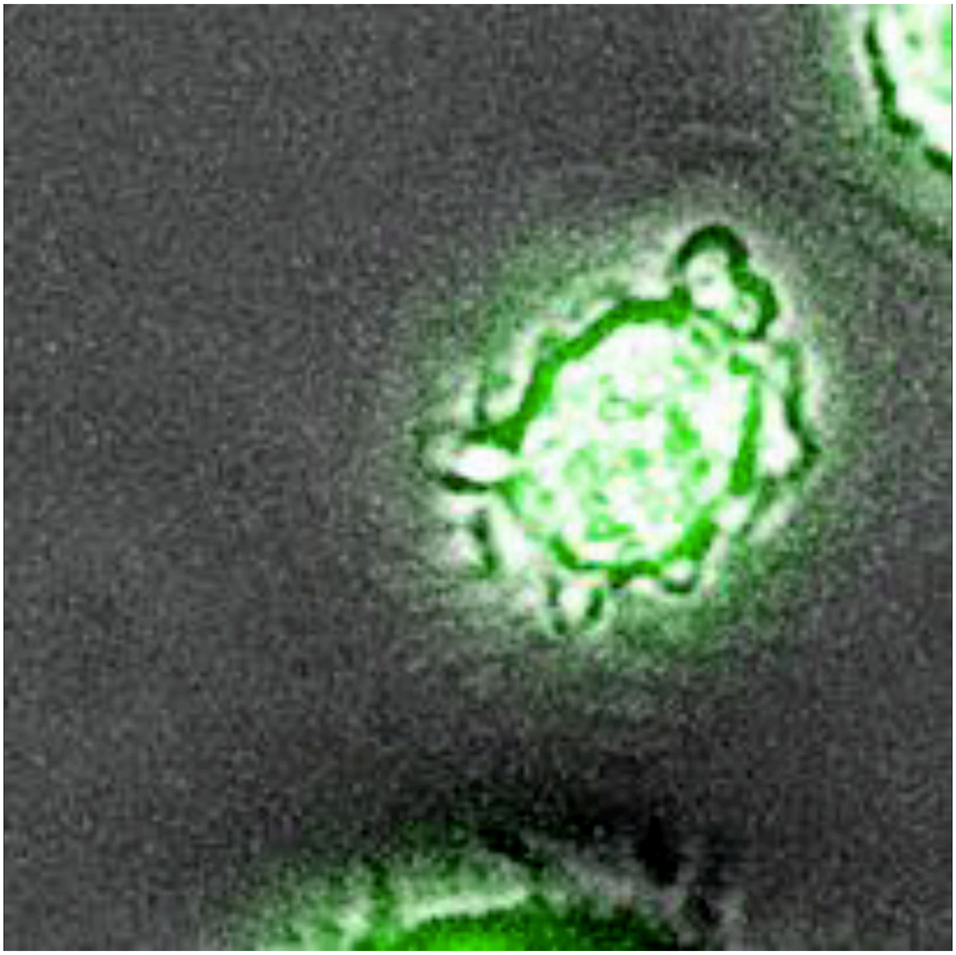

Supplementary Video 4: Taxol-treated cell undergoing mitosis. HeLa-FUCCI cells were synchronized, treated with taxol, and imaged by live time-lapse microscopy as described for Video S1.

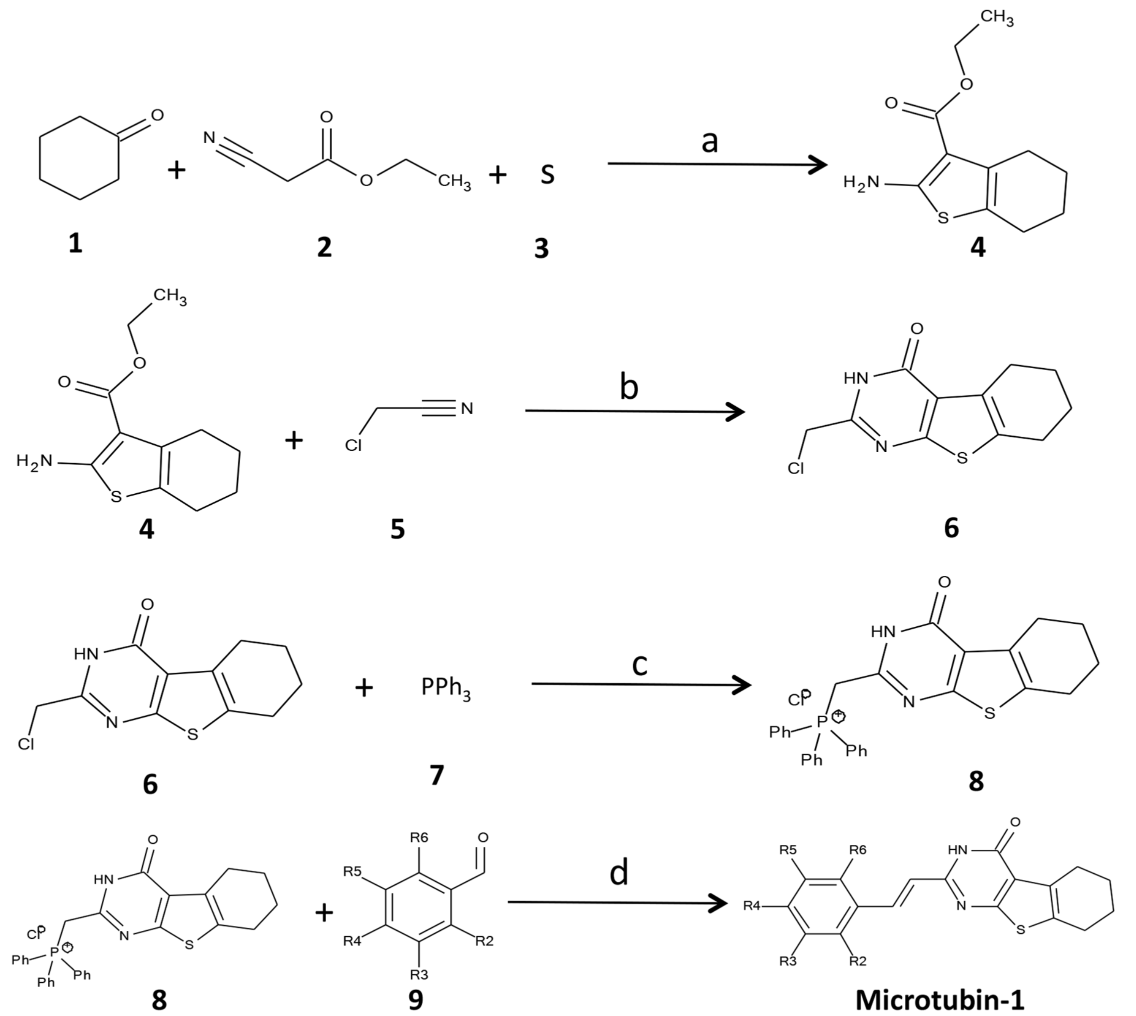

Microtubin-1

9a: R_3=R_5=R_6=H, R_2=R_4=F
9b:R_3=R_4=R_6=H, R_2=R_5=F
9c: R_2=R_3=R_5=R_6=H, R_4=F
9d: R_2=R_4=R_5=R_6=H, R_3=OMe
9e: R_2=R_3=R_5=R_6=H, R_4=SMe
9f: R_2=R_5=R_6=H, R_3=OMe, R_4=OEt
9g: R_2=R_4=R_5=R_6=H, R_3=Me
9h: R_2=R_3=R_5=R_6=H, R_4=OMe
9i: R_2=R_3=R_5=R_6=H, R_4=Br
9j: R_2=R_5=R_6=H, R_3=R_4=F
9k: R_2=R_5=R_6=H, R_3=R_4=OMe
9l: R_2=R_3=R_5=R_6=H, R_4=OEt
9m: R_2=R_3=R_5=R_6=H, R_4=Me

Microtubin-2: R_3=R_5=R_6=H, R_2=R_4=F
Microtubin-3:R_3=R_4=R_6=H, R_2=R_5=F
Microtubin-4: R_2=R_3=R_5=R_6=H, R_4=F
Microtubin-5: R_2=R_4=R_5=R_6=H, R_3=Ome
Microtubin-6: R_2=R_3=R_5=R_6=H, R_4=Sme
Microtubin-7: R_2=R_5=R_6=H, R_3=OMe, R_4=Oet
Microtubin-8: R_2=R_4=R_5=R_6=H, R_3=Me
Microtubin-9: R_2=R_3=R_5=R_6=H, R_4=Ome
Microtubin-10: R_2=R_3=R_5=R_6=H, R_4=Br
Microtubin-11: R_2=R_5=R_6=H, R_3=R_4=F
Microtubin-12: R_2=R_5=R_6=H, R_3=R_4=Ome
Microtubin-13: R_2=R_3=R_5=R_6=H, R_4=Oet
Microtubin-14: R_2=R_3=R_5=R_6=H, R_4=Me

Reagents and conditions: (a) Morpholine (20.4 g, 0.234 mol) , EtOH, 50C; (b) HCl, 3hr, 1,4-dioxane, 50C; (c)toluene, 8hr; (d) Na_2CO_3 (0.8mL, 10%), MeOH, rt.

Supplementary Figure 1: Workflow for the synthesis of Microtubin-1 and its analogues.

A

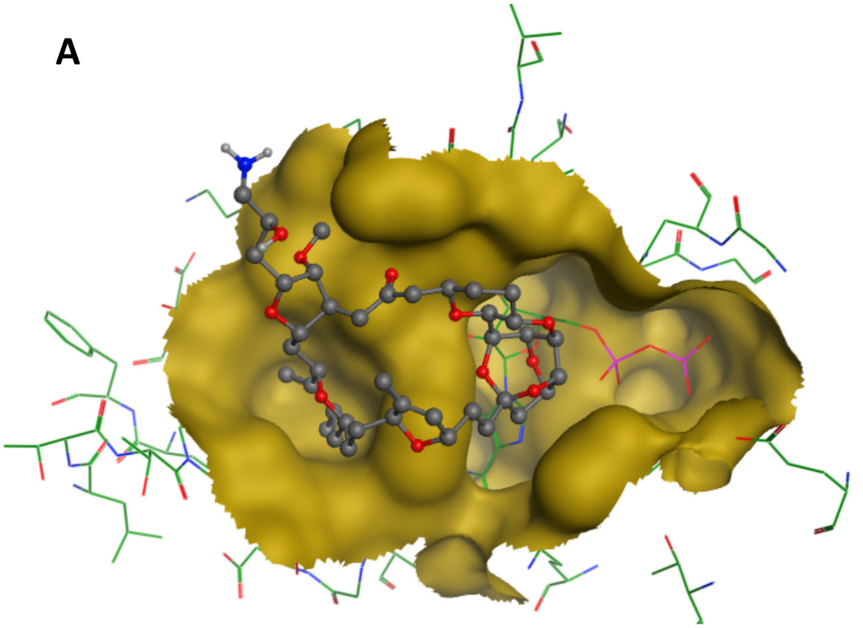

B

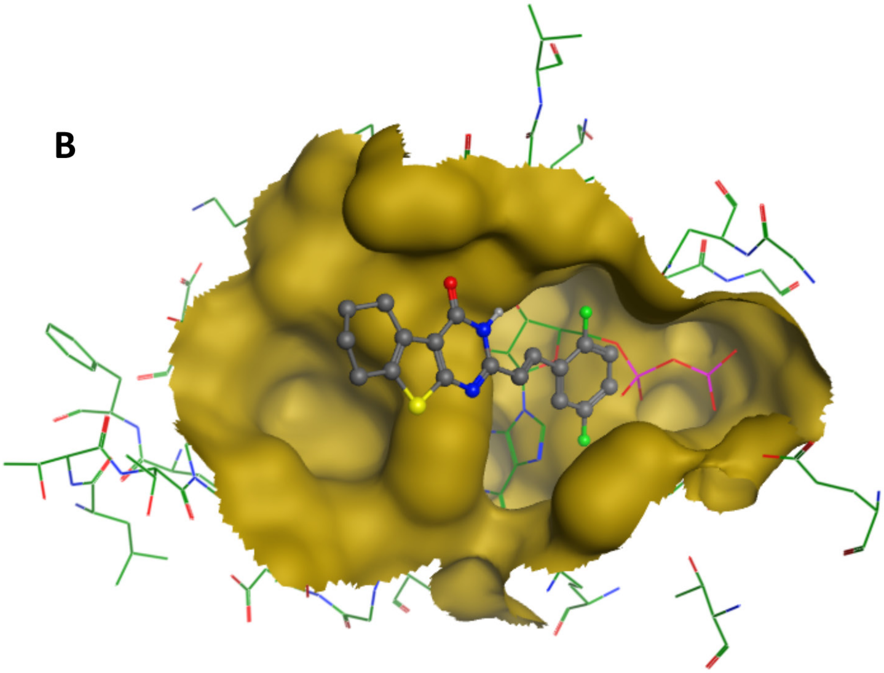

Supplementary Figure 2: Molecular Docking of Eribulin and Microtubin-3 to the Eribulin receptor pocket. **(A)**, Eribulin was docked into its tubulin receptor pocket using the Eribulin-tubulin co-crystal structure (PDB ID: 5JH7) and MOE software. **(B)**, the placement of Microtubin-3 into the Eribulin site yielded a significantly lower docking score (London dG score: -6.55) than that of Eribulin (London dG score: -13.47).

Supplementary Table 1: 397 compounds with varied functional group additions to the core scaffold of Microtubin-1

See Supplementary File 1

Supplementary Table 2: 38 phenyl ring derivatives based on the Topliss scheme for aromatic ring optimization of Microtubin-1

See Supplementary File 2

Supplementary Table 3: 26 Microtubin-1 drug-like compounds tested in SAR analysis

See Supplementary File 3
